# Environmental triggers for photosynthetic protein turnover determine the optimal nitrogen distribution and partitioning in the canopy

**DOI:** 10.1093/jxb/ery308

**Published:** 2018-08-17

**Authors:** Yi-Chen Pao, Tsu-Wei Chen, Dany Pascal Moualeu-Ngangue, Hartmut Stützel

**Affiliations:** Institute of Horticultural Production Systems, Leibniz Universität Hannover, Hannover, Germany

**Keywords:** Functional partitioning, light, mechanistic model, nitrogen reallocation, nitrogen supply, optimal, photosynthetic acclimation

## Abstract

Plants continually adjust the photosynthetic functions in their leaves to fluctuating light, thereby optimizing the use of photosynthetic nitrogen (*N*_ph_) at the canopy level. To investigate the complex interplay between external signals during the acclimation processes, a mechanistic model based on the concept of protein turnover (synthesis and degradation) was proposed and parameterized using cucumber grown under nine combinations of nitrogen and light in growth chambers. Integrating this dynamic model into a multi-layer canopy model provided accurate predictions of photosynthetic acclimation of greenhouse cucumber canopies grown under high and low nitrogen supply in combination with day-to-day fluctuations in light at two different levels. This allowed us to quantify the degree of optimality in canopy nitrogen use for maximizing canopy carbon assimilation, which was influenced by *N*_ph_ distribution along canopy depth or *N*_ph_ partitioning between functional pools. Our analyses suggest that *N*_ph_ distribution is close to optimum and *N*_ph_ reallocation is more important under low nitrogen. *N*_ph_ partitioning is only optimal under a light level similar to the average light intensity during acclimation, meaning that day-to-day light fluctuations inevitably result in suboptimal *N*_ph_ partitioning. Our results provide insights into photoacclimation and can be applied to crop model improvement.

## Teaser

A mechanistic model of photosynthetic protein turnover explains leaf acclimation to light and nitrogen availabilities and suggests that suboptimal photosynthetic nitrogen partitioning is due to daily light fluctuations.

## Introduction

Acclimation of leaf traits to fluctuating environments is a key mechanism to maximize fitness (Walters, 2005; Athanasiou *et al.*, 2010). To maximize canopy carbon gain, dynamic modifications of photosynthetic traits to track heterogeneous light distribution within the canopy are crucial (Retkute *et al.*, 2015), especially for herbaceous species with a continuously leaf-forming nature (Niinemets *et al.*, 2015). One of the most important strategies in photoacclimation is to maintain efficient utilization of limited resources in the photosynthetic apparatus, e.g. nitrogen, by continuous modifications of (i) between-leaf distribution along the canopy depth and (ii) within-leaf partitioning between photosynthetic functions according to local light availability (Evans, 1989).

Vertical nitrogen distribution in response to light has been intensively studied (Hirose and Werger, 1987; Werger and Hirose, 1991; Anten *et al.*, 1995; Dreccer *et al.*, 2000; Moreau *et al.*, 2012; Hikosaka *et al.*, 2016). Nitrogen distribution was reported to closely follow the light gradient and thus approach its optimum in wheat stands (Dreccer *et al.*, 2000). However, this relationship has not been found in other studies (Moreau *et al.*, 2012; Hikosaka *et al.*, 2016). In fact, many studies demonstrated that nitrogen distribution failed to track the within-canopy light gradient optimally due to a delay in nitrogen reallocation in the lower canopy layer and an underinvestment in the upper layer (Field, 1983; Evans, 1993; Hollinger, 1996; Hirose *et al.*, 1997; Meir *et al.*, 2002; Wright *et al.*, 2006; Hikosaka, 2016). This discrepancy between optimum and reality could be explained by physiological limitations and the cost of nitrogen reallocation (Hikosaka, 2016; Kitao *et al.*, 2018) or might result from incorrect predictions. In some cases (e.g. Hikosaka, 2014; Kitao *et al.*, 2018), the optimal nitrogen distribution that followed the within-canopy light gradient estimated by the Beer–Lambert law was predicted to be extremely high in the upper canopy, which might not be biologically reachable. This could result from the oversimplification of models in three aspects: (i) neglecting the effects of variations in the structural characteristics, e.g. leaf elevation angle (Falster and Westoby, 2003), on light interception of the leaves; (ii) neglecting age-dependent modifications and limitations during leaf development and ageing (Niinemets *et al.*, 2015; Niinemets, 2016); and (iii) assuming a linear relationship between photosynthetic capacity and photosynthetic nitrogen per unit leaf area instead of considering photoacclimation in functional nitrogen partitioning.

Optimizing functional partitioning within the leaf is of great importance because it improves carbon gain by enhancing photosynthetic nitrogen use efficiency (PNUE; Zhu *et al.*, 2010). Photosynthetic rate is determined by the limited rate of ribulose 1,5-bisphosphate (RuBP) carboxylation and RuBP regeneration in the photosynthetic machinery (Farquhar *et al.*, 1980). Besides driving photosynthesis, light also triggers fine adjustments in nitrogen investment between (i) RuBP carboxylation (Rubisco), (ii) RuBP regeneration (electron transport), and (iii) light harvesting functions (Yamori *et al.*, 2010; Trouwborst *et al.*, 2011; Vialet-Chabrand *et al.*, 2017). The capability and significance of photoacclimation in functional nitrogen partitioning were empirically addressed in both light-demanding and shade-tolerant species (Evans, 1993; Hikosaka and Terashima, 1996; Pons and Anten, 2004; Hikosaka, 2005; Trouwborst *et al.*, 2011). Recently, with a modelling approach, it was predicted that a decreasing investment in the light harvesting function can increase canopy PNUE (Song *et al.*, 2017). However, genetic and physiological controls of photoacclimatory processes by environmental triggers are still not described mechanistically.

The degree of acclimation under a given environment is limited by the previous environmental conditions (Walters, 2005; Niinemets *et al.*, 2006) along with continuous age-dependent modifications in physiological traits (Niinemets, 2016). This emphasizes that static models, which do not consider the dynamics of plant growth and environmental fluctuations, may not be sufficiently precise in predicting acclimation behavior. Prieto *et al.* (2012) proposed an empirical model describing the combined effects of leaf age and light on leaf nitrogen economics for a grapevine canopy and demonstrated that the mean daily light integral over the previous 10 d explained 73% of the variation in nitrogen per unit leaf area. Since environmental acclimation and developmental (genetic control of leaf ageing) acclimation are regulated distinctively (Athanasiou *et al.*, 2010), it is possible to integrate internal (age) and external (environment) triggers into a mechanistic model for better understanding of the developmental and environmental effects on photosynthetic acclimation.

Acclimation processes in leaf functioning are regulated by constant updates of protein content as a result of protein turnover, driven by the concurrent actions of degradation and synthesis (Li *et al.*, 2017). In growing leaves, photosynthetic proteins account for the highest cost in protein turnover (Li *et al.*, 2017). At the expense of energy, protein turnover is necessary for adjusting protein levels in line with external triggers. It was experimentally shown that leaf Rubisco content increased with light (Yamori *et al.*, 2010) and nitrogen supply level (Yamori *et al.*, 2011*a*) and exhibited an evolution with leaf age that could be interpreted by Rubisco turnover (Suzuki *et al.*, 2001; Ishimaru *et al.*, 2001; Irving and Robinson, 2006). Based on the concept of protein turnover, Thornley (1998) proposed a mechanistic model predicting reasonable dynamics of photosynthetic acclimation at the leaf level. We refined this model to describe the dynamics of different photosynthetic nitrogen pools and to quantify the developmental and environmental effects of light and nitrogen availabilities on leaf acclimation. The optimality of nitrogen distribution and partitioning at the canopy scale was evaluated by integrating this model into a multi-layer model considering the structural characteristics of a cucumber canopy. This aims (i) to test whether the protein turnover can be a mechanistic explanation of the photosynthetic acclimation under dynamic environmental conditions; and (ii) to understand the regulatory mechanism of environmental triggers on the degree of optimality at the canopy level in terms of maximizing PNUE and canopy carbon assimilation, which can be considered as an indicator of the general fitness of the plants.

## Materials and methods

### Modelling the dynamics of photosynthetic protein turnover

Photosynthetic nitrogen (*N*_ph_, mmol N m^−2^) is defined as biologically active nitrogen in the proteins involved in photosynthetic functions, i.e. carboxylation, electron transport and light harvesting. Leaf *N*_ph_ is calculated as the sum of nitrogen in the carboxylation pool (*N*_V_), electron transport pool (*N*_J_) and light harvesting pool (*N*_C_, Trouwborst *et al.*, 2011):

$$N_{\text{ph}}=N_{\text{V}}+N_{\text{J}}+N_{\text{C}}$$ (1)

where *N*_V_ includes only Rubisco and represents the nitrogen investment in carboxylation capacity, *N*_J_ includes the electron transport chain, photosystem II core and Calvin cycle enzymes other than Rubisco, and *N*_C_ includes the photosystem I core and light harvesting complexes I and II. Functional pools *N*_V_, *N*_J_, and *N*_C_ are estimated from the maximum carboxylation rate (*V*_cmax_, μmol CO_2_ m^−2^ s^−1^), maximum electron transport (*J*_max_, μmol e^−^ m^−2^ s^−1^) and leaf chlorophyll (*Chl*, mmol Chl m^−2^), respectively (Buckley *et al.*, 2013):

$$N_{\text{V}}=V_{\text{cmax}}/\chi_{\text{V}}$$ (2a)

$$N_{\text{J}}=J_{\text{max}}/\chi_{\text{J}}$$ (2b)

$$N_{\text{C}}=\left(Chl-N_{\text{J}}\times \chi_{\text{CJ}}\right)/\chi_{\text{C}}$$ (2c)

where χ_V_ (μmol CO_2_ mmol^−1^ N s^−1^) is the carboxylation capacity per unit Rubisco nitrogen, and χ_J_ (μmol e^−^ mmol^−1^ N s^−1^) is the electron transport capacity per unit electron transport nitrogen. χ_CJ_ (mmol Chl mmol^−1^ N) and χ_C_ (mmol Chl mmol^−1^ N) are the conversion coefficients for chlorophyll per electron transport nitrogen and per light harvesting component nitrogen, respectively. Photosynthetic nitrogen partitioning fraction of a pool *X* (*p*_*X*_) is determined as the ratio of nitrogen in the pool *X* (*N*_*X*_, mmol N m^−2^) to *N*_ph_:

$$p_{X}=N_{X}/N_{\text{ph}}$$ (3)

The rate of change of *N*_*X*_ is determined by the instantaneous protein synthesis rate [*S*_*X*_(*t*), mmol N m^−2^ °Cd^−1^] and degradation rate [*D*_*X*_(*t*), mmol N m^−2^ °Cd^−1^] of the corresponding enzymes and protein complexes at a given leaf age (*t*, °Cd):

$$\text{d}N_{X}/\text{d}t=S_{X}\left(t\right)-D_{X}\left(t\right)$$ (4)

Protein synthesis as an age-dependent and zero-order process (Li *et al.*, 2017) is described by a logistic function and independent of the current *N*_*X*_ state:

$$S_{X}\left(t\right)=2S_{\text{max},X}/\left[1+\text{exp}\left(t\times t_{\text{d},X}\right)\right]$$ (5)

where *S*_max,*X*_ (mmol N m^−2^ °Cd^−1^) is the maximum protein synthesis rate of *N*_*X*_ that occurs at the early stage of leaf development (Supplementary Fig. S1 at *JXB* online). The constant *t*_d,*X*_ (°Cd^−1^) describes the relative decreasing rate of the protein synthesis over time (see Table 1 for the coefficients used in the protein turnover model). At age of 1/*t*_d,*X*_, *S*_*X*_ reduces to 53.8% of *S*_max,*X*_.

**Table 1.** List of coefficients used in the protein turnover model for photosynthetic nitrogen pools, carboxylation pool *N*_V_, electron transport pool *N*_J_, and light harvesting pool *N*_C_

Description	Coefficient	Unit	Pool *N*_V_	Pool *N*_J_	Pool *N*_C_
Degradation constant [Eq. (6)]	*D*_r_	°Cd^−1^	0.0195	0.0195	0.0091
Increase rate constant of *S*_max_ with *I*_Ld_ [Eq. (7)]	*k*_I_	mmol N m^2^ ground d m^−2^ LA °Cd^−1^ mol^−1^ photon	0.173	0.130	0.234
Michaelis–Menten constant relating *N*_S_ to *S*_max_ [Eq. (8)]	*k*_N_	mM	0.536	0.420	0.316
Potential maximum synthesis rate [Eq. (7)]	*S*_mm_	mmol N m^−2^ °Cd^−1^	1.122	0.852	0.248
Decreasing constant of synthesis rate [Eq. (5)]	*t*_d_	°Cd^−1^	0.001	0.002	0.001

The coefficients were estimated from the growth chamber experiment. Model variables and other coefficients are listed in Tables 2 and 3.

The degradation rate *D*_*X*_ is governed by first-order kinetics (Verkroost and Wassen, 2005; Li *et al.*, 2017) with a degradation constant *D*_r,*X*_ (°Cd^−1^):

$$D_{X}\left(t\right)=D_{r,X}\times N_{X}\left(t\right)$$ (6)

The variable *S*_max,*X*_ in Eq. (5) is a function of daily light interception (*I*_Ld_, mol photons m^−2^ d^−1^):

$$S_{\text{max},X}=\left[S_{\text{mm},X}\times k_{\text{I},X}\times I_{\text{Ld}}/\left(S_{\text{mm},X}+k_{\text{I},X}\times I_{\text{Ld}}\right)\right]\times r_{\text{N},X}$$ (7)

where *S*_mm,*X*_ (mmol N m^−2^ °Cd^−1^) is the potential maximum protein synthesis rate and *k*_I,*X*_ is the rate constant describing the increase of *S*_max,*X*_ with *I*_Ld_. The factor *r*_N,*X*_ increases with nitrogen level in the nutrient solution (*N*_S_, mM) by a Michaelis–Menten constant, *k*_N,*X*_ (mM):

$$r_{\text{N},X}=N_{\text{S}}/\left(k_{\text{N},X}+N_{\text{S}}\right)$$ (8)

### Modelling leaf photosynthesis

Photosynthetic parameters *V*_cmax_, *J*_max_, and *Chl* were estimated from functional nitrogen pools *N*_V_, *N*_J_, and *N*_C_, using Eq. (2a–c). The net photosynthetic rate (*A*, μmol CO_2_ m^−2^ s^−1^) is defined as the minimum of RuBP carboxylation-limited (*A*_c_, mmol CO_2_ m^−2^ s^−1^) and RuBP regeneration-limited (*A*_j_, mmol CO_2_ m^−2^ s^−1^) net photosynthetic rate (Farquhar *et al.*, 1980):

$$A=\text{min}\;\left(A_{\text{c}},A_{\text{j}}\right)$$ (9a)

$$A_{\text{c}}=V_{\text{c}}\times \left(C_{\text{c}}-\Gamma^{\text{*}}\right)/\left[C_{\text{c}}+K_{\text{c}}\left(1+O/K_{\text{o}}\right)\right]-R_{\text{d}}$$ (9b)

$$A_{\text{j}}=J\times \left(C_{\text{c}}-\Gamma^{\text{*}}\right)/\left(4C_{\text{c}}+8\Gamma^{\text{*}}\right)-R_{\text{d}}$$ (9c)

where *C*_c_ (μmol CO_2_ mol^−1^) is the chloroplastic CO_2_ concentration, Γ* (μmol CO_2_ mol^−1^) is the CO_2_ compensation point in the absence of dark respiration, *K*_c_ (μmol CO_2_ mol^−1^) and *K*_o_ (mmol O_2_ mol^−1^) are Michaelis–Menten constants of Rubisco for CO_2_ and O_2_, respectively, *O* (mmol O_2_ mol^−1^) is the O_2_ concentration at the site of carboxylation, *V*_c_ (μmol CO_2_ m^−2^ s^−1^) is carboxylation rate, and *J* (μmol e^−^ m^−2^ s^−1^) is electron transport rate. Daytime respiration rate *R*_d_ (μmol CO_2_ m^−2^ s^−1^) is assumed to vary with *t* and the mean *I*_Ld_ during the previous 4 d (*I*_Ld4d_):

$$R_{\text{d}}\left(t\right)=R_{\text{max}}\times I_{\text{Ld}4\text{d}}\times \text{exp}\left(-R_{\text{g}}\times I_{\text{Ld}4\text{d}}\times t\right)+R_{\text{m}}\times I_{\text{Ld}4\text{d}}\times t$$ (10)

where *R*_max_ (μmol CO_2_ d mol^−1^ photons s^−1^) relates *I*_Ld4d_ to the maximum *R*_d_, *R*_g_ (m^2^ d °Cd^−1^ mol^−1^ photons) influences the decrease in the growth respiration, and *R*_m_ (μmol CO_2_ d °Cd^−1^ mol^−1^ photons s^−1^) affects the increase in the maintenance respiration with *t*.

*V*_c_ and *J* are calculated from *V*_cmax_ and *J*_max_, respectively, depending on the photosynthetic photon flux density (PPFD) incident on the leaf (*I*_Lc_, µmol photons m^–2^ s^–1^) according to Qian *et al.* (2012) and Ögren and Evans (1993), respectively:

$$V_{\text{c}}=V_{\text{cmax}}\left\{0.31+\frac{0.69}{1+\text{exp}\left[-0.009\times \left(I_{\text{Lc}}-500\right)\right]}\right\}$$ (11)

$$J=\left\{\phi \times \alpha \times I_{\text{Lc}}+J_{\text{max}}-{\left[\begin{array}{l} {\left(\phi \times \alpha \times I_{\text{Lc}}+J_{\text{max}}\right)}^{2}\\ -4\theta \times J_{\text{max}}\times \phi \times \alpha \times I_{\text{Lc}} \end{array}\right]}^{0.5}\right\}/\left(2\theta \right)$$ (12)

where ϕ (µmol e^–^ µmol photons^−1^) is the conversion efficiency of photons to *J*, and θ (unitless) is a constant convexity factor describing the response of *J* to *I*_Lc_. Leaf absorptance (α, unitless) is related to *Chl* (Evans, 1993):

α=Chl/(Chl+0.076) (13)

Chloroplastic CO_2_ concentration depends on the steady-state of stomatal conductance (*g*_sc_, mol CO_2_ m^−2^ s^−1^) and mesophyll conductance (*g*_m_, mol CO_2_ m^−2^ s^−1^) to CO_2_:

$$C_{\text{c}}=C_{\text{a}}-A\times \left[\left(g_{\text{sc}}+g_{\text{m}}\right)/\left(g_{\text{sc}}\times g_{\text{m}}\right)\right]$$ (14)

where *C*_a_ (μmol CO_2_ mol^−1^) is atmospheric CO_2_ concentration, and *g*_sc_ is calculated with species-specific constants of stomatal conductance, *g*_0_ and *g*_1_ (Chen *et al.*, 2014), and leaf-to-air vapor pressure deficit (*D*, kPa, Medlyn *et al.*, 2011):

$$g_{\text{sc}}=g_{0}+\left(1+g_{1}/\sqrt{D}\right)\times A/C_{\text{a}}$$ (15)

Mesophyll conductance is expressed as a log-normal function of *t* (Chen *et al.*, 2014), where *g*_m_ first increases during leaf development and decreases during ageing (Flexas *et al.*, 2008):

$$g_{\text{m}}=g_{\text{mmax}}\times \text{exp}\left\{-0.5\times {\left[\text{ln}\left(t/t_{\text{gm}}\right)/v_{\text{gm}}\right]}^{2}\right\}$$ (16)

where *t*_gm_ is the *t* when the maximum *g*_m_ (*g*_mmax_, mol CO_2_ m^−2^ s^−1^) occurs and *v*_gm_ is the standard deviation of the curve; *g*_mmax_ is linearly related to *N*_ph_, since a similar relationship has been reported for *C*_3_ plants (e.g. Yamori *et al.*, 2011*a*):

$$g_{\text{mmax}}=r_{\text{gm}}\times N_{\text{ph}}+r_{\text{gm}0}$$ (17)

where *r*_gm_ (mol CO_2_ mmol^−1^ N s^−1^) describes the rate of increase of *g*_mmax_ in relation to *N*_ph_, and *r*_gm0_ (mol CO_2_ m^−2^ s^−1^) is the minimum *g*_mmax_.

The steady-state *A*_c_ was solved analytically with Eqs (9b), (14), and (15), and *A*_j_ with Eqs (9c), (14), and (15), following Moualeu‐Ngangue *et al.* (2016). Model variables and coefficients are listed in Tables 1–3.

**Table 2.** List of model input and output variables

Description	Variable	Unit	Equation	Type
Net photosynthetic rate	*A*	μmol CO_2_ m^−2^ s^−1^	9a	Output
RuBP carboxylation-limited *A*	*A*_c_	μmol CO_2_ m^−2^ s^−1^	9b	Output
RuBP regeneration-limited *A*	*A*_j_	μmol CO_2_ m^−2^ s^−1^	9c	Output
Leaf absorptance	α	—	13	Output
Atmospheric CO_2_ concentration	*C*_a_	μmol CO_2_ mol^−1^	—	Input
Chloroplastic CO_2_ concentration	*C*_c_	μmol CO_2_ mol^−1^	14	Output
Leaf chlorophyll per unit area	*Chl*	mmol m^−2^	2c	Output
Leaf-to-air vapor pressure deficit	*D*	kPa	—	Input
Protein degradation rate of N pool *X*	*D*_*X*_	°Cd^−1^	6	Output
Factor for creating variation in N distribution	*f*_d_	—	18	Input
Factor for creating variation in N partitioning	*f*_p_	—	19	Input
Mesophyll conductance to CO_2_	*g*_m_	mol CO_2_ m^−2^ s^−1^	16	Output
Maximum *g*_m_	*g*_mmax_	mol CO_2_ m^−2^ s^−1^	17	Output
Stomatal conductance to CO_2_	*g*_sc_	mol CO_2_ m^−2^ s^−1^	15	Output
PPFD at leaf	*I*_Lc_	μmol photons m^−2^ s^−1^	—	Input
Daily photosynthetic photon integral at leaf	*I*_Ld_	mol photons m^−2^ d^−1^	—	Input
Mean *I*_Ld_ during the last 4 d	*I*_Ld4d_	mol photons m^−2^ d^−1^	—	Input
Electron transport rate	*J*	μmol e^−^ m^−2^ s^−1^	12	Output
Maximum electron transport rate	*J*_max_	μmol e^−^ m^−2^ s^−1^	2b	Output
Leaf area	LA	m^2^	—	Input
Total leaf photosynthetic N content in the canopy	*N*_canopy_	mmol N	—	Output
Leaf photosynthetic N content	*N*_leaf_	mmol N	—	Output
Leaf photosynthetic N per unit area	*N*_ph_	mmol N m^−2^	1	Output
N concentration of nutrient solution	*N*_s_	mM	—	Input
Concentration of N pool *X*	*N*_*X*_	mmol N m^−2^	4	Output
Concentration of N pool of light harvesting	*N*_C_	mmol N m^−2^	4	Output
Concentration of N pool of electron transport	*N*_J_	mmol N m^−2^	4	Output
Concentration of N pool of carboxylation	*N*_V_	mmol N m^−2^	4	Output
Partitioning fraction of N pool *X*	*p*_*X*_	—	3	Output
Daytime respiration rate in the absence of photorespiration	*R*_d_	μmol CO_2_ m^−2^ s^−1^	10	Output
Reduction factor of protein synthesis depending on N availability	*r*_N_	—	8	Output
Maximum protein synthesis rate	*S*_max_	mmol N m^−2^ °Cd^−1^	7	Output
Protein synthesis rate of N pool *X*	*S*_*X*_	mmol N m^−2^ °Cd^−1^	5	Output
Leaf age	*t*	°Cd	—	Input
Carboxylation rate	*V*_c_	μmol CO_2_ m^−2^ s^−1^	11	Output
Maximum carboxylation rate	*V*_cmax_	μmol CO_2_ m^−2^ s^−1^	2a	Output

**Table 3.** List of model coefficients

Description	Coefficient	Unit	Value (SE)	Reference
Conversion coefficient of chlorophyll per light harvesting N	χ_C_	mmol Chl mmol^−1^ N	0.03384	Buckley *et al.* (2013)
Conversion coefficient of chlorophyll per electron transport N	χ_CJ_	mmol Chl mmol^−1^ N	4.64 × 10^–4^	Buckley *et al.* (2013)
Conversion coefficient of electron transport capacity per electron transport N	χ_J_	μmol e^−^ mmol^−1^ N s^−1^	9.48	Buckley *et al.* (2013)
Conversion coefficient of carboxylation capacity per Rubisco N	χ_V_	μmol CO_2_ mmol^−1^ N s^−1^	4.49	Buckley *et al.* (2013)
Minimum *g*_sc_	*g*_0_	mol CO_2_ m^−2^ s^−1^	0.009	Chen *et al.* (2014)
Species-specific coefficient of *g*_sc_	*g*_1_	—	3.51	Chen *et al.* (2014)
CO_2_ compensation point in the absence of dark respiration	Γ*	μmol CO_2_ mol^−1^	43.02	Singsaas *et al.* (2004)
Michaelis–Menten constant of Rubisco for CO_2_	*K*_c_	μmol CO_2_ mol^−1^	404	Chen *et al.* (2014)
Michaelis–Menten constant of Rubisco for O_2_	*K*_o_	mmol O_2_ mol^−1^	278	Chen *et al.* (2014)
O_2_ concentration at the site of carboxylation	*O*	mmol O_2_ mol^−1^	210	Chen *et al.* (2014)
Coefficient relating *N*_ph_ to *g*_mmax_	*r*_gm_	mol CO_2_ mmol^−1^ N s^−1^	1.64 × 10^–3^ (5.27 × 10^–4^)	—
Minimum *g*_mmax_	*r*_gm0_	mol CO_2_ m^−2^ s^−1^	0.140 (0.0345)	—
Coefficient related to the decrease in *R*_d_ by growth respiration	*R*_g_	m^2^ d °Cd^−1^ mol^−1^ photon	4.16 × 10^–4^ (4.52 × 10^–5^)	—
Coefficient related to the increase in *R*_d_ by maintenance respiration	*R*_m_	μmol CO_2_ d °Cd^−1^ mol^−1^ photons s^−1^	1.88 × 10^–4^ (1.61 × 10^–5^)	—
Coefficient relating *I*_Ld_ to maximum *R*_d_	*R*_max_	μmol CO_2_ μmol^−1^ photons s^−1^	0.308 (0.028)	—
Conversion efficiency of photons to *J*	ϕ	µmol e^–^ µmol^−1^ photons	0.340 (2.5 × 10^–3^)	—
Convexity coefficient	θ	—	0.7	Chen *et al.* (2014)
Leaf age when *g*_mmax_ occurs	*t*_gm_	°Cd	121 (8.1)	—
Standard deviation of the dependence of *g*_m_–*t* curve	*v*_gm_	—	0.860 (0.063)	—

Standard errors (SE) are indicated in parentheses.

### Growth chamber experiment to investigate the dynamics of photosynthetic protein turnover

Cucumber (*Cucumis sativus* ‘Aramon’, Rijk Zwaan, De Lier, The Netherlands) plants were grown in two experiments at the Institute of Horticultural Production Systems, Leibniz Universität Hannover, Germany (latitude 52.4°N).

One growth chamber experiment was conducted from 21 October to 9 December 2016 with factorial combinations of three light and three nitrogen supply levels to parameterize the photosynthetic protein turnover model (see below). Cucumber seeds were sown in rock-wool cubes (36 × 36 × 40 mm) on 5 October. Eight days later, seedlings were transplanted to larger rock-wool cubes (10 × 10 × 6.2 cm) for another 8 d until the second true leaves appeared (leaf length ≥3 cm). Plants were transferred into 25 litre plastic containers (one plant per container) on 21 October and cultivated hydroponically with a 12 h light period and 24 °C day/20 °C night air temperature. Three nitrogen levels, 9.6, 4.6 and 2.3 mM, were supplied using Ca(NO_3_)_2_ and Ferty Basisdünger 1 (Planta GmbH, Regenstauf, Germany, 5.2 mM K, 1.3 mM P, 0.82 mM Mg in working solution). Nutrient solution was replaced weekly and adjusted to pH 6.0–6.5 two times a week. Three constant light conditions with daily photosynthetic photon integrals (DPI) of 28.9, 14.2, and 4.4 mol photons m^−2^ d^−1^ were provided using metal halide lamps. Four plants were grown under each treatment combination. Three leaves per plant (between leaf ranks four to eight, counted acropetally) were maintained horizontally and well exposed to incoming light using custom-made leaf holders, while the rest of the shoot was trained downward to avoid mutual shading. Gas exchange (see below) and relative chlorophyll content (SPAD-502; Minolta Camera, Japan) were measured at different thermal ages of the leaves, ranging from 45 °Cd to 558 °Cd, calculated by subtracting a base temperature of 10 °C (Savvides *et al.*, 2016) from mean daily air temperature around the leaf. Air temperature was recorded continuously using data loggers (Tinytag; Gemini Data Loggers, Chichester, UK). After gas exchange measurements, leaves were harvested for leaf area and nitrogen analyses.

### Greenhouse experiment to evaluate optimality of nitrogen distribution and partitioning

One greenhouse experiment was carried out from 4 April to 12 May 2017 under two light regimes and two nitrogen supply levels to evaluate the model performance and to collect input data for optimality analyses. Seeds were sown on 14 March and transplanted to larger rock-wool cubes on 22 March. After the third true leaves had appeared, plants were transferred onto rock-wool slabs on 4 April with plant density of 1.33 plants m^−2^ and supplied with two nitrogen concentrations, 10 mM (high nitrogen, HN) and 2.5 mM (low nitrogen, LN), by drip irrigation using the same fertilizers as described in the growth chamber experiment. During the experimental period, average nitrogen supply was calculated from the nitrogen concentration in the nutrient supply and rock-wool slabs, which was 8.2 and 2.0 mM for HN and LN, respectively. Plants were grown under either high light (HL) or low light (LL) regimes. The southern half of the greenhouse was unshaded as the HL regime. The LL regime was created in the northern half of the greenhouse by shading nets to reduce incoming light from top and sides, where PPFD was reduced on average to *ca*. 40% of that under HL (38 ± 1.3% under sunny and 42 ± 0.2% under cloudy condition). Average DPI above the canopy was 21.4 and 8.5 mol photons m^−2^ d^−1^ for HL and LL, respectively, during the experimental period. DPI during the experimental period was recorded by the weather station located above the greenhouse. An average light transmittance of 49.8% through the greenhouse structure was applied (39.2% on a sunny day and 60.4% on a cloudy day). Air temperature in the middle canopy was recorded continuously using data loggers and was significantly higher under HL (0.5 °Cd per day). Gas exchange measurements and harvests were conducted at four time points on 21 April, 28 April, 5 May, and 12 May at two different canopy layers with two replications. Leaf age at measurement ranged from 77 to 414 °Cd. Leaf elevation angle was obtained by a 3D digitizer (Fastrak; Polhemus, Colchester, VT, USA) according to Chen *et al.* (2014). Leaves were harvested after gas exchange measurements to determine leaf area index (LAI, m^2^ m^−2^).

### Gas exchange measurements and estimation of photosynthetic parameters

Light-saturated net photosynthetic rate under PPFD of 1300 µmol photons m^−2^ s^−1^ (*A*_1300_, μmol CO_2_ m^−2^ s^−1^) and light response curves were measured using a portable photosynthesis system (LI-6400XT; Li-Cor Inc., Lincoln, NE, USA). All measurements were carried out under sample CO_2_ 400 µmol mol^−1^, leaf temperature 25 °C and relative humidity 55–65%. *R*_d_ was estimated from the linear portion of the light response curve (Kok, 1948). *V*_cmax_ was estimated using the one-point method (Wilson *et al.*, 2000; De Kauwe *et al.*, 2016), and *J*_max_ and ϕ by least squares fitting to a non-rectangular hyperbola (Ögren and Evans, 1993). Mesophyll conductance was estimated using the variable *J* method (Harley *et al.*, 1992). Chlorophyll fluorescence was measured using the multiphase flash approach (Loriaux *et al.*, 2013) following Moualeu‐Ngangue *et al.* (2017).

### Nitrogen analyses and photosynthetic nitrogen partitioning estimation

Leaf samples obtained in the growth chamber experiment were freeze-dried and ground into a fine powder for nitrogen analyses. Total leaf nitrogen was analysed using the Kjeldahl method (Nelson and Sommers, 1980). Leaf chlorophyll was extracted with 96% ethanol and analysed colorimetrically (Lichtenthaler, 1987). Relationships between relative chlorophyll content (SPAD) and *Chl* were determined (Supplementary Fig. S2) for estimating *Chl* in the greenhouse experiment.

### Model parameterization

The differential equations (4)–(6) were solved and the coefficients were quantified using R (version 3.3.0; R Foundation for Statistical Computing, Vienna, Austria) by using the packages ‘deSolve’ and ‘DEoptim’, which minimizes the sums of squares of the residuals between observations and simulations. The data obtained in the growth chamber experiment were used for the parameterization. *D*_r,*X*_ and *t*_d,*X*_ were first quantified for each pool using data of all treatments. With the determined values of *D*_r,*X*_ and *t*_d,*X*_, *S*_max,*X*_ was then quantified for each treatment. *S*_mm,*X*_, *k*_I,*X*_, and *k*_N,*X*_ were determined from *S*_max,*X*_ [Eqs (7) and (8)] by least squares fitting in SigmaPlot (version 11.0, Systat software GmbH, Erkrath, Germany) as well as the influences of *t* and *I*_Ld_ on *R*_d_ [Eq. (10)] and *g*_m_ [Eqs (16) and (17)].

### Dynamic leaf photosynthetic nitrogen simulation and model evaluation

Daily environmental information during the experimental period (Supplementary Fig. S3) and the canopy information obtained at the four harvests, including age and area of each leaf, were used as input to simulate photosynthetic nitrogen per unit leaf area (*N*_ph_, mmol N m^−2^), photosynthetic nitrogen per leaf (*N*_leaf_, mmol N) and total leaf photosynthetic nitrogen content of the canopy (*N*_canopy_, mmol N). First, leaf elevation angle of each leaf and LAI were simulated empirically depending on *t* (Supplementary Fig. S4). Second, for each time step, the daily light interception *I*_Ld_ at the leaf was calculated and used in Eq. (7) to simulate protein turnover. Light interception was calculated by the Beer–Lambert law (Monsi and Saeki, 1953) with a light extinction coefficient of 0.695 and adjusted by the cosine of leaf elevation angle. For model evaluation, root mean squared deviation (RMSD) and accuracy (%) were determined for photosynthetic parameters, *N*_ph_, and *p*_*X*_ predictions following Kahlen and Stützel (2011).

### Simulating daily canopy carbon assimilation

Daily canopy carbon assimilation during daytime (DCA, mol CO_2_ d^−1^) was simulated using greenhouse canopy characteristics obtained at the last harvest as input (Supplementary Table S1; Supplementary Fig. S5). Leaf-to-air vapor pressure deficit (*D*) 1.2 kPa and *C*_a_ 400 μmol CO_2_ mol^−1^ were used in all simulations, similar to the environmental conditions during the gas exchange measurements. Scenarios with different DPI levels were defined for simulating DCA. Up to six DPI levels were taken as relative to the average DPI during acclimation (aDPI) to simulate the influence of day-to-day DPI fluctuation on DCA. To simulate DCA, diurnal PPFD above the canopy was simulated for a given DPI level with a time step of 0.1 h by a simple cosine bell function (Kimball and Bellamy, 1986) with 14.4 h day length.

### Modifying photosynthetic nitrogen distribution and partitioning

To evaluate the effects of between-leaf distribution and within-leaf partitioning of *N*_ph_ on DCA, a distribution factor *f*_d_ was introduced into Eq. (5) to create variations in the rate of protein synthesis, and a partitioning factor *f*_p,*X*_ was introduced into Eq. (7) to create variations in the maximum protein synthesis rate of different functional pools:

$$S_{X}\left(t\right)=2S_{\text{max},X}/\left[1+\text{exp}\left(t\times t_{\text{d},X}\times f_{\text{d}}\right)\right]$$ (18)

$$S_{\text{max},X}=\left[\begin{array}{l} S_{\text{mm},X}\times f_{\text{p},X}\times k_{\text{I},X}\times I_{\text{Ld}}\\ /\left(S_{\text{mm},X}\times f_{\text{p},X}+k_{\text{I},X}\times I_{\text{Ld}}\right) \end{array}\right]\times r_{\text{N},X}$$ (19)

A control condition was defined with *f*_d_=1 and *f*_p,*X*_=1, when all coefficients in the synthesis process (Table 1) remained unmodified. Increasing *f*_d_ accelerates the decrease in the rate of protein synthesis and enhances acropetal *N*_ph_ reallocation. An increase in *f*_p,*X*_ results in a higher rate of synthesis of *N*_*X*_ and increases the partitioning to pool *X*. A modified partitioning pattern that maximized DCA was identified as optimal for several DPI levels, and the optimal values of *f*_p,*X*_ were determined using the package ‘DEoptim’ in R. The change in DCA caused by modified distribution or optimal partitioning of *N*_ph_ was compared with the control conditions. The ratios between optimal and control partitioning fractions of each pool *X*, as well as the contributions of daily leaf carbon assimilation (DLA) to the DCA increase were calculated along the canopy depth.

## Results

### Mechanistic model aims to quantify the environmental effects of light and nitrogen availabilities and developmental effects on photosynthetic protein turnover

**Fig. 1.**
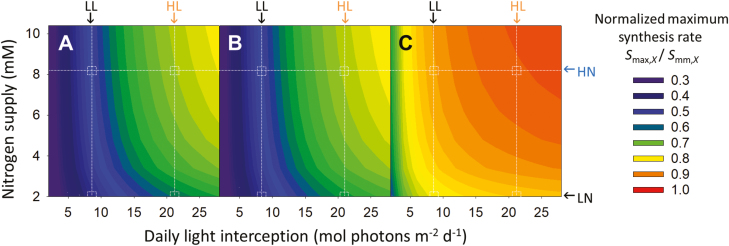
Simulated effects of daily light interception (*I*_Ld_, mol photons m^−2^ d^−1^) and nitrogen supply level in the nutrient solution (*N*_S_, mM) on maximum protein synthesis rate (*S*_max,*X*_) in Eq. (7) using coefficients from Table 1, of (A) the carboxylation, (B) the electron transport and (C) the light harvesting pools. The colors denote the normalized maximum protein synthesis rate, which is *S*_max,*X*_ normalized by the potential maximum protein synthesis rate (*S*_mm,*X*_) in Eq. (7). The data obtained in the growth chamber experiment were used for the parameterization. The arrows above and beside the figures indicate the corresponding average environmental conditions in the greenhouse experiment: high light (HL) 21.4 mol photons m^−2^ d^−1^; low light (LL) 8.5 mol photons m^−2^ d^−1^; high nitrogen (HN) 8.2 mM; low nitrogen (LN) 2.0 mM.

In the model, we assume that photosynthetic protein turnover is under genetic and environmental control. The genetic control is characterized by the potential maximum protein synthesis rate *S*_mm_, coefficient *t*_d_, and protein degradation constant, *D*_r_. The coefficient *t*_d_ affects the decrease in the rate of synthesis, and *D*_r_ contributes to the degradation rate, which together influence the developmental effect on protein turnover dynamics. The low value of *t*_d_ (0.001–0.002 °Cd^−1^, Table 1) suggests that the influence of ageing appears rather late in the leaf lifespan under a constant light environment. The coefficient *D*_r_ was found to be the same for the carboxylation pool (*N*_V_) and the electron transport pool (*N*_J_), while the light harvesting pool (*N*_C_) had a lower *D*_r_ (Table 1). The genotypic sensitivities to light and nitrogen availabilities are characterized by *k*_I_ and *k*_N_, respectively. Collectively, *S*_mm_, *k*_I_, and *k*_N_ determine the maximum protein synthesis rate *S*_max_ in Eq. (7). When light was increased 2.5-fold (from LL to HL), *S*_max_ increased by 50% in *N*_V_ and *N*_J_, and by 10% in *N*_C_, while nitrogen level had less influence on *S*_max_ (<10%), which only occurred under low nitrogen concentration (<3.5 mM) and the higher light intensity (Fig. 1), showing that light had a major control of *S*_max_. *N*_C_ had the highest *k*_I_ (Table 1); consequently, *S*_max,C_ approached saturation at lower light intensity than *S*_max,V_ and *S*_max,J_ (Fig. 1). *S*_max,V_ and *S*_max,J_ were well coordinated in response to light and nitrogen level (Fig. 1A, B), but the higher *k*_I_ and *k*_N_ of *N*_V_ (Table 1) suggested that *N*_V_ synthesis is more sensitive to the variation in light and nitrogen availabilities than *N*_J_.

### Effects of light and nitrogen availabilities on maximal protein synthesis rate explain the dynamics of photosynthetic acclimation

**Fig. 2.**
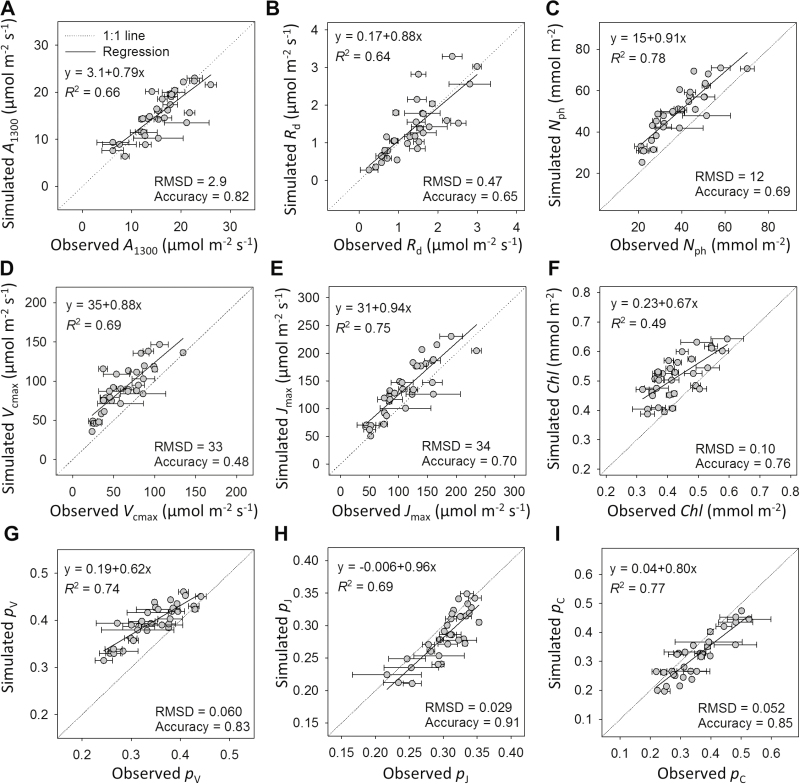
Comparisons between simulated and observed leaf photosynthetic parameters. (A) Light-saturated net photosynthetic rate under PPFD 1300 µmol photons m^−2^ s^−1^ (*A*_1300_, µmol CO_2_ m^−2^ s^−1^); (B) daytime respiration rate (*R*_d_, µmol CO_2_ m^−2^ s^−1^); (C) leaf photosynthetic nitrogen (*N*_ph_, mmol N m^−2^); (D) maximum carboxylation rate (*V*_cmax_, µmol CO_2_ m^−2^ s^−1^); (E) maximum electron transport rate (*J*_max_, µmol e^−^ m^−2^ s^−1^); (F) chlorophyll (*Chl*, mmol Chl m^−2^); (G) partitioning fraction of the carboxylation pool (*p*_V_); (H) partitioning fraction of the electron transport pool (*p*_J_); and (I) partitioning fraction of the light harvesting pool (*p*_C_). The observed data were obtained in the greenhouse experiment. The dotted grey lines are one-to-one lines. Root mean squared deviation (RMSD) and accuracy of the predictions are shown (see Materials and methods).

**Fig. 3.**
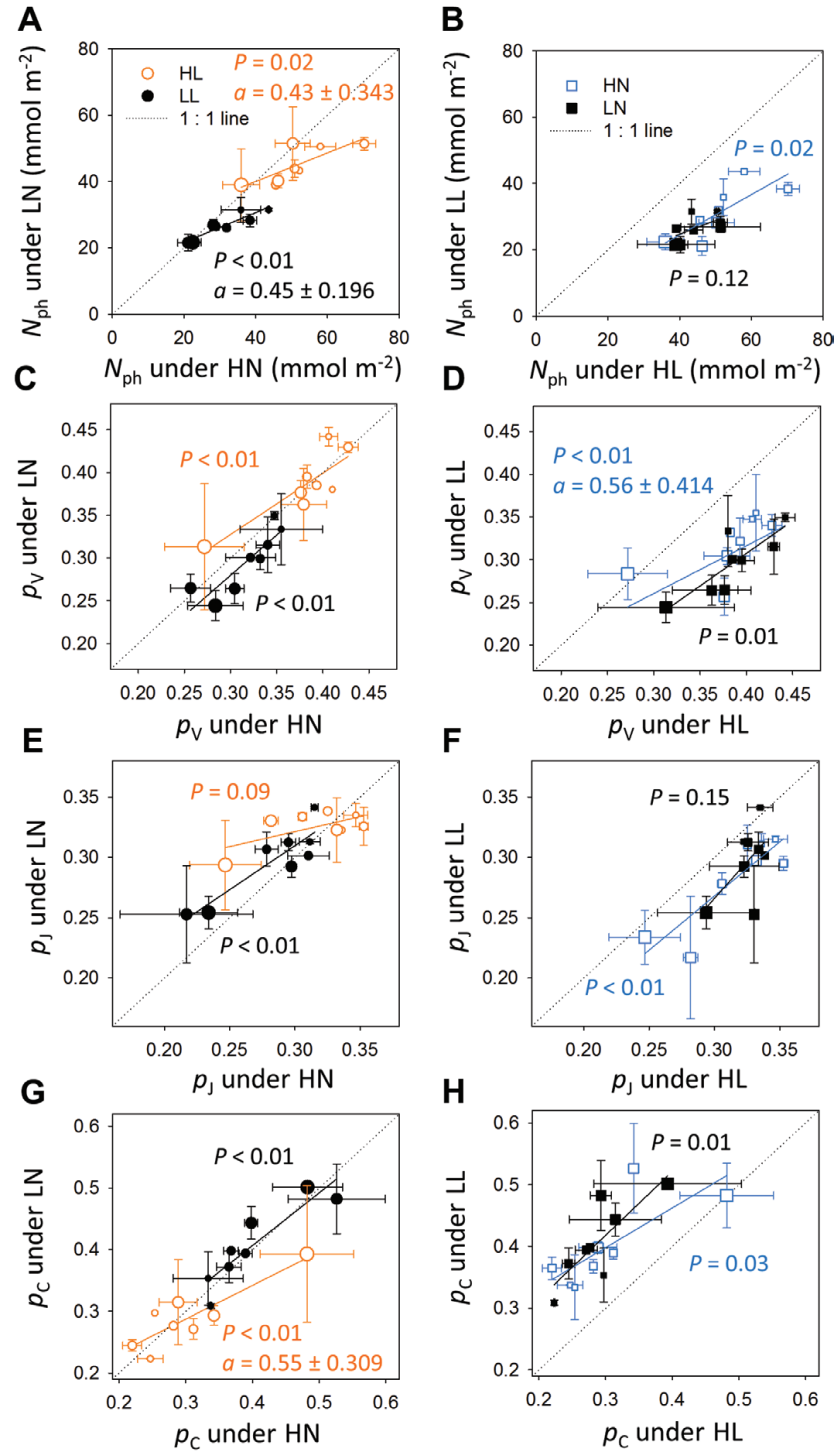
Comparisons of leaf photosynthetic nitrogen (*N*_ph_, mmol N m^−2^; A, B), partitioning fractions of the carboxylation pool (*p*_V_; C, D), the electron transport pool (*p*_J_; E, F), and the light harvesting pool (*p*_C_; G, H) between high and low nitrogen supply (HN and LN, respectively; A, C, E, G) and between high and low light conditions (HL and LL, respectively; B, D, F, H). Each point represents the measurements in the greenhouse experiment obtained from a comparable canopy layer. The orange open circles indicate leaves grown under HL, the black closed circles indicate LL, the blue open squares indicate HN and the black closed squares indicate LN. The size of the circles increases with leaf age, ranging from 77 °Cd to 414 °Cd. The solid lines show the linear regression *y*=*ax* + *b*. The *P* values of the slope *a* are shown. The values of *a* are specified with 95% confidence intervals when they are significantly different from 1. The dotted grey lines are one-to-one lines.

We evaluated the model using a greenhouse experiment, where leaves grown under combinations of two light regimes (HL and LL) and two nitrogen levels (HN and LN) were measured in two canopy layers weekly for four consecutive weeks. The model predicted leaf photosynthetic characteristics with high accuracy (70–91%, Fig. 2) and a trend of photosynthetic acclimation (Supplementary Fig. S6) similar to the experimental observations (Fig. 3), except for slight overestimations in photosynthetic nitrogen (*N*_ph_, Fig. 2C), carboxylation pool (Fig. 2D, G), and chlorophyll (Fig. 2F).

Photosynthetic acclimation in the greenhouse canopies as influenced by the interplay between light, nitrogen level and leaf age was examined (Fig. 3). Light had positive effects on *N*_ph_ (Fig. 3A), the partitioning fractions of *N*_V_ (*p*_V_, Fig. 3C) and *N*_J_ (*p*_J_, Fig. 3E) but negative effects on the partitioning fraction of *N*_C_ (*p*_C_, Fig. 3G). This negative effect of light on *p*_C_ can be explained by the high *k*_I_ of *N*_C_ (Table 1), which leads to an saturation of *S*_max,C_ under lower light (Fig. 1). The changes in *N*_ph_, *p*_V_, *p*_J_, and *p*_C_ with leaf age were similar to those with light (Fig. 3) due to the association in the gradients of age and light.

In comparison with HN, *N*_ph_ under LN was significantly lower in the young leaves but similar in the old leaves (Fig. 3A). In the greenhouse, young leaves developed under high light intensity, which increased the sensitivity of *S*_max_ to nitrogen level (Fig. 1). During the simultaneous increase in leaf age and mutual shading, the effects of nitrogen supply on *S*_max_ became less prevalent (Fig. 1). Nitrogen level had less influence on functional partitioning (Fig. 3C, E, G) than light (Fig. 3D, F, H).

### Photosynthetic nitrogen distribution is close to optimum and the effect of nitrogen reallocation is more prominent under limited nitrogen availability

The influence of *N*_ph_ distribution pattern along the canopy depth on daily canopy carbon assimilation (DCA, mol CO_2_ d^−1^) was evaluated by introducing a distribution factor *f*_d_ to create variations in the rate of protein synthesis. In our model, protein synthesis and degradation rates determined simultaneously (i) total leaf photosynthetic nitrogen content of the canopy (*N*_canopy_, mmol N), (ii) *N*_ph_ distribution in the canopy, and (iii) *N*_ph_ partitioning fractions of pools *X* (*p*_*X*_) in the leaf. Thus, it was impossible to modify single elements while maintaining the other two constant. Increasing *f*_d_ led to a faster reduction of *N*_ph_ during leaf ageing and more acropetal *N*_ph_ reallocation. However, it also reduced *N*_canopy_ and tended to increase *p*_C_ (data not shown). Therefore, to obtain the leaf photosynthetic nitrogen content (*N*_leaf,*i*_, mmol N in leaf *i*) with comparable *N*_canopy_, simulated *N*_leaf,*i*_ with *f*_d_=*n* (denoted as *N′*_leaf,*i*_) was adjusted proportionally to the ratio between *N*_canopy_ calculated with *f*_d_=1 and with *f*_d_=*n*:

$$N_{\text{leaf},i}\left(f_{\text{d}}=n\right)=N{'}_{\text{leaf},i}\left(f_{\text{d}}=n\right)\times \left[N_{\text{canopy}}\left(f_{\text{d}}=1\right)/N_{\text{canopy}}\left(f_{\text{d}}=n\right)\right]$$ (20a)

*p*_*X*,*i*_ was set equal to the control value:

$$p_{X,i}=N_{X,i}\left(f_{\text{d}}=1\right)/N_{\text{ph},i}\left(f_{\text{d}}=1\right)$$ (20b)

**Fig. 4.**
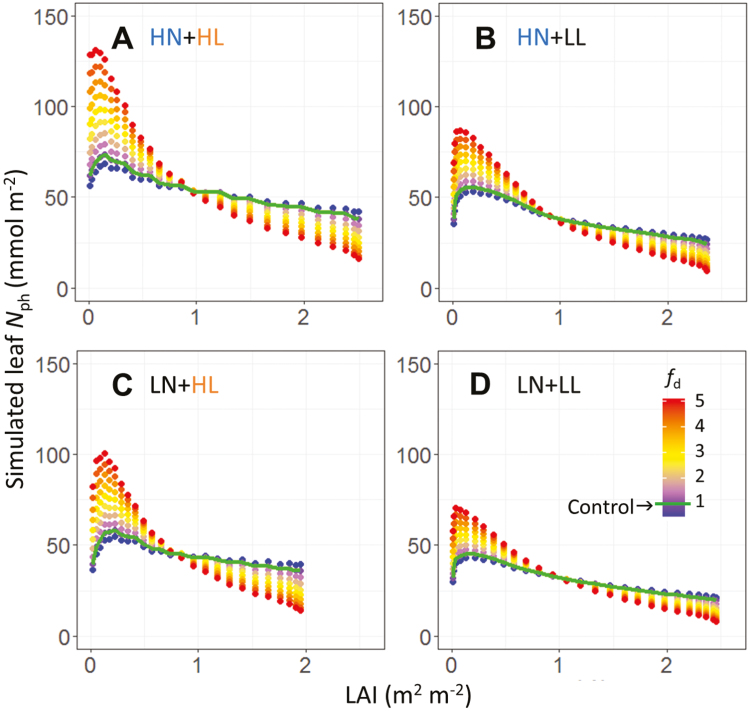
Leaf photosynthetic nitrogen (*N*_ph_, mmol N m^−2^) distributions along the canopy depth, characterized by leaf area index (LAI, m^2^ m^−2^). Variations in nitrogen distribution were created using a distribution factor *f*_d_ ranging from 0.5 to 5.0 at intervals of 0.5 in Eq. (18) under different growth conditions. (A) High nitrogen and high light (HN+HL); (B) high nitrogen and low light (HN+LL); (C) low nitrogen and high light (LN+HL); (D) low nitrogen and low light (LN+LL). Simulated control *N*_ph_ distributions (*f*_d_=1) are indicated by the green lines.

These adjustments assured the same amount of *N*_canopy_ among the distribution patterns. The factor *f*_d_ was varied between 0.5 and 5.0 at intervals of 0.5 in the simulation, which gave values of *N*_ph_ comparable to those measured in cucumber leaves (22–135 mmol N m^−2^; Fig. 4). Canopy *N*_ph_ distributions with enhanced acropetal reallocation were created by increasing *f*_d_ (Fig. 4; Supplementary Fig. S7). In general, the distribution of *N*_ph_ corresponded to the vertical light distribution except in the expanding leaves in the upper canopy, and the *N*_ph_ distribution with light was steeper under LL (Supplementary Fig. S7).

**Fig. 5.**
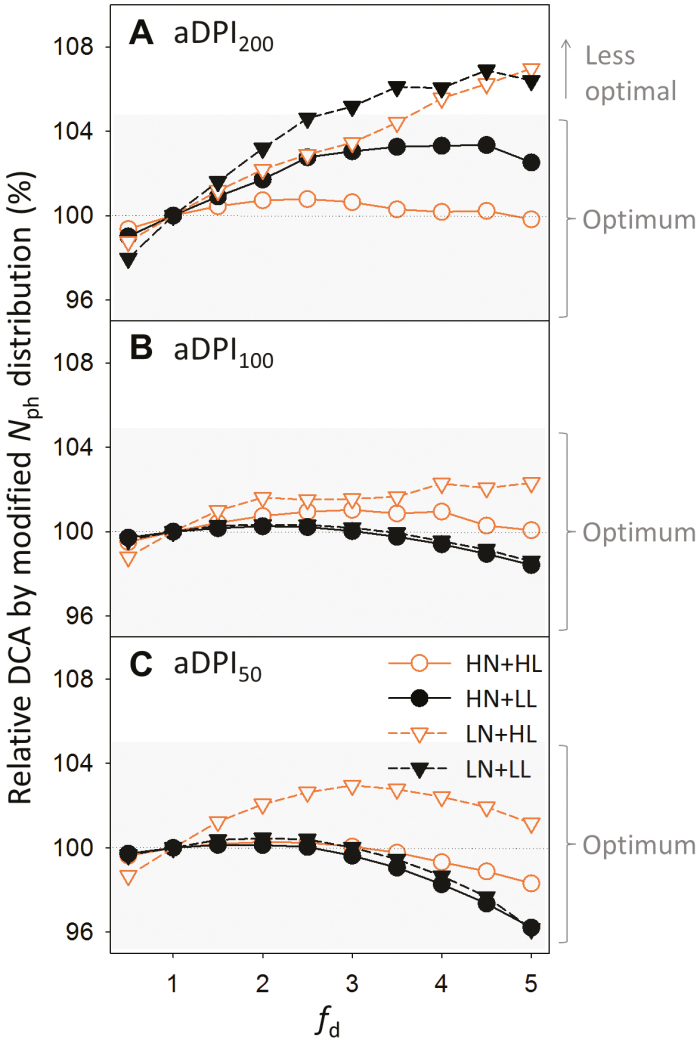
Effects of photosynthetic nitrogen (*N*_ph_) distributions with different values of *f*_d_ (Fig. 4) on daily canopy carbon assimilation (DCA) under different daily photosynthetic photon integrals (DPI, mol photons m^−2^ d^−1^) relative to average DPI during acclimation (aDPI). (A) Two-fold aDPI (aDPI_200_); (B) aDPI (aDPI_100_); (C) half aDPI (aDPI_200_). Acropetal *N*_ph_ reallocation increases with *f*_d_. Plants grown under high nitrogen and high light (HN+HL, orange open circles), under high nitrogen and low light (HN+LL, black closed circles), under low nitrogen and high light (LN+HL, orange open triangles), and under low nitrogen and low light (LN+LL, black closed triangles) are compared under given DPI. The relative change in DCA was calculated by dividing the DCA obtained with a given *N*_ph_ distribution by the DCA obtained with the control *N*_ph_ distribution (*f*_d_=1) under same DPI. A change within ±5% (grey shading) is considered insignificant.

To simulate the natural fluctuations in light between days, three light levels representing 200% (aDPI_200_), 100% (aDPI_100_) and 50% (aDPI_50_) of average DPI during acclimation (aDPI) were used in the DCA simulation. Under aDPI_100_ and aDPI_50_, enhancing acropetal *N*_ph_ reallocation did not significantly increase DCA (<5%), suggesting that *N*_ph_ distribution was optimal under constant and decreasing DPI (Fig. 5B, C). More acropetal reallocation did not improve the optimality in *N*_ph_ distribution in terms of maximizing DCA since a large proportion of leaf area was located in the middle-lower to lower canopy (Supplementary Fig. S5). However, enhancing *N*_ph_ reallocation resulted in an increase in DCA by 7% under LN at aDPI_200_ (Fig. 5A), indicating that acropetal *N*_ph_ reallocation was more important under LN than HN.

It was observed that *N*_ph_ was more overestimated in the older leaves than in the younger ones (Fig. 2C), which indicated that our model tended to underestimate the acropetal *N*_ph_ reallocation when scaling up from leaf to canopy level. In order to maintain a constant light environment for the measured leaves in the growth chamber experiment, leaves younger than the sampled leaves were trained downward and their light interception, together with their nitrogen demand, was inevitably reduced; therefore, the model coefficients were obtained from the leaves with limited nitrogen reallocation. However, underestimating acropetal *N*_ph_ reallocation would not affect our result that *N*_ph_ distribution was close to optimum.

### Suboptimal nitrogen partitioning is due to daily light fluctuation

**Fig. 6.**
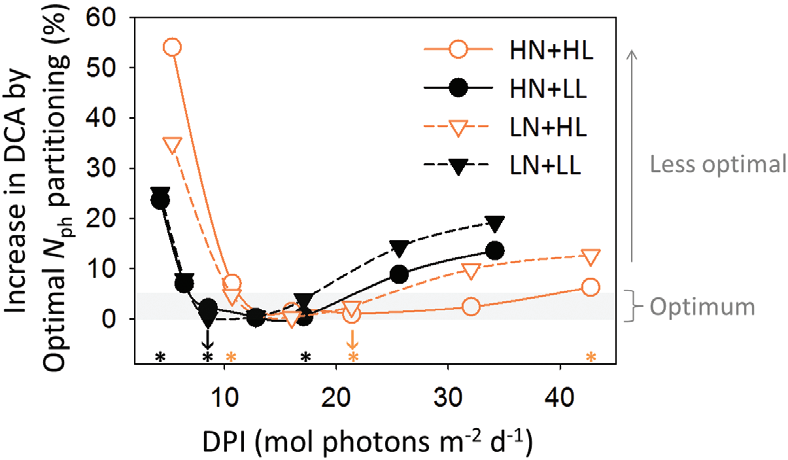
Increase in daily canopy carbon assimilation (DCA) by optimizing photosynthetic nitrogen (*N*_ph_) partitioning for different growth conditions under various daily photosynthetic photon integrals (DPI, mol photons m^−2^ d^−1^). The increase in DCA was the DCA with the optimal partitioning under a given DPI in comparison with the control partitioning [*f*_p,*X*_=1 in Eq. (19)]. An increase less than 5% (grey shading) is considered insignificant. The average DPI during acclimation (aDPI) is indicated by the orange arrow for HL (21.4 mol photons m^−2^ d^−1^) and by the black arrow for LL (8.5 mol photons m^−2^ d^−1^). The asterisks indicate the scenarios compared in Figs 7, 8 and Table 4 with 50%, 100% and 200% aDPI. The symbols and colors used here are the same as those in Fig. 5.

To find the optimal within-leaf *N*_ph_ partitioning between functions, the potential maximal protein synthesis rate for pool *X* was modified by a factor *f*_p,*X*_, ranging from 0.2 to 2.0. Increasing *f*_p,*X*_ resulted in higher protein synthesis rates, but it also increased *N*_canopy_ and the proportion of nitrogen distributed in the upper canopy. After simulating nitrogen partitioning with a modified *f*_p,*X*_, *N*_leaf_ of each leaf was re-assigned to their control values that were obtained with *f*_p,*X*_=1. Partitioning patterns with maximal DCA at six DPI levels (25–400% aDPI) were identified as optimal and the maximal DCA was compared with control DCA (Fig. 6). The increase in DCA by optimal partitioning was insignificant (<5%) when DPI was close to aDPI (indicated by the arrows in Fig. 6). This suggested the ability of plants to maximize DCA by optimizing *N*_ph_ partitioning to aDPI. *N*_ph_ partitioning deviated further from optimum when DPI diverged from aDPI (Fig. 6). Therefore, strong day-to-day light fluctuation induced suboptimality in *N*_ph_ partitioning and led to lower PNUE.

**Table 4.** Increase in the daily canopy carbon assimilation (DCA) by optimized photosynthetic nitrogen distribution or partitioning under various daily photosynthetic photon integrals (DPI, mol photons m^−2^ d^−1^) for canopies grown under different conditions

Growth condition	Light level		Control DCA	Increase in DCA (%) by optimized	
	aDPI level (%)	DPI	(mol CO_2_ d^−1^)	Distribution	Partitioning
HN+HL	200	42.7	0.5467	<5%	6.3%
	100	21.4	0.3217	<5%	<5%
	50	10.7	0.1368	<5%	7.1%
HN+LL	200	17.1	0.2554	<5%	<5%
	100	8.5	0.1195	<5%	<5%
	50	4.3	0.0259	<5%	23.6%
LN+HL	200	42.7	0.4011	7.0%	12.7%
	100	21.4	0.2653	<5%	<5%
	50	10.7	0.1221	<5%	<5%
LN+LL	200	17.1	0.2261	6.9%	<5%
	100	8.5	0.1108	<5%	<5%
	50	4.3	0.0215	<5%	25.0%

Average DPI during acclimation (100% aDPI), 200% and 50% aDPI were tested. The increase in DCA for plants grown under the combinations of high nitrogen (HN), high light (HL), low nitrogen (LN), and low light (LL) was calculated by comparing the DCA between optimal and control distribution or partitioning.

**Fig. 7.**
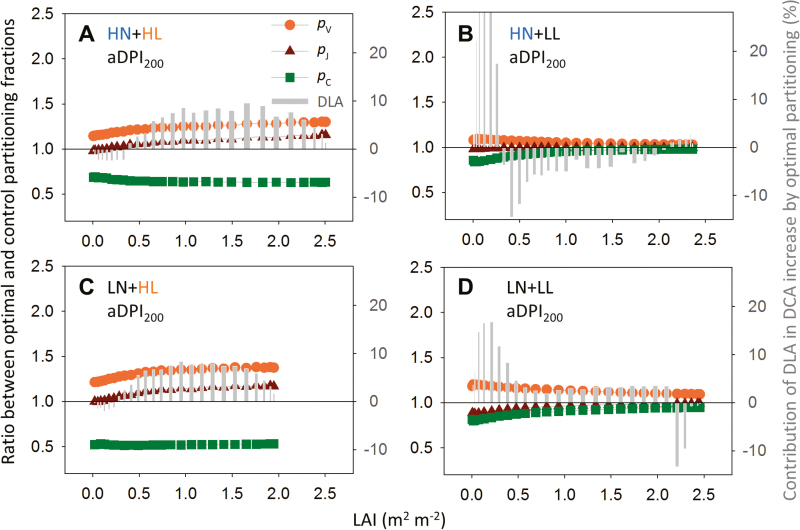
Ratio between optimal and control partitioning fractions (optimal *p*_*X*_/control *p*_*X*_) of the carboxylation pool (*p*_V_, orange circles), the electron transport pool (*p*_J_, red triangles), the light harvesting pool (*p*_C_, green squares), and contributions of daily leaf carbon assimilation (DLA) to the daily canopy carbon assimilation (DCA) increase by optimal partitioning (grey bars, right *y*-axis) along the canopy depth [leaf area index (LAI) m^2^ m^−2^] under 200% average daily photosynthetic photon integral during acclimation (aDPI_200_) for plants grown under (A) high nitrogen and high light (HN+HL), (B) high nitrogen and low light (HN+LL), (C) low nitrogen and high light (LN+HL), (D) low nitrogen and low light (LN+LL) conditions. Photosynthetic nitrogen partitioning is close to optimum for HN+LL and LN+LL under aDPI_200_, which corresponds to a DPI of 42.7 and 17.1 mol photons m^−2^ d^−1^ for HL and LL, respectively. See Table 4 for the increase in DCA by the optimal partitioning.

**Fig. 8.**
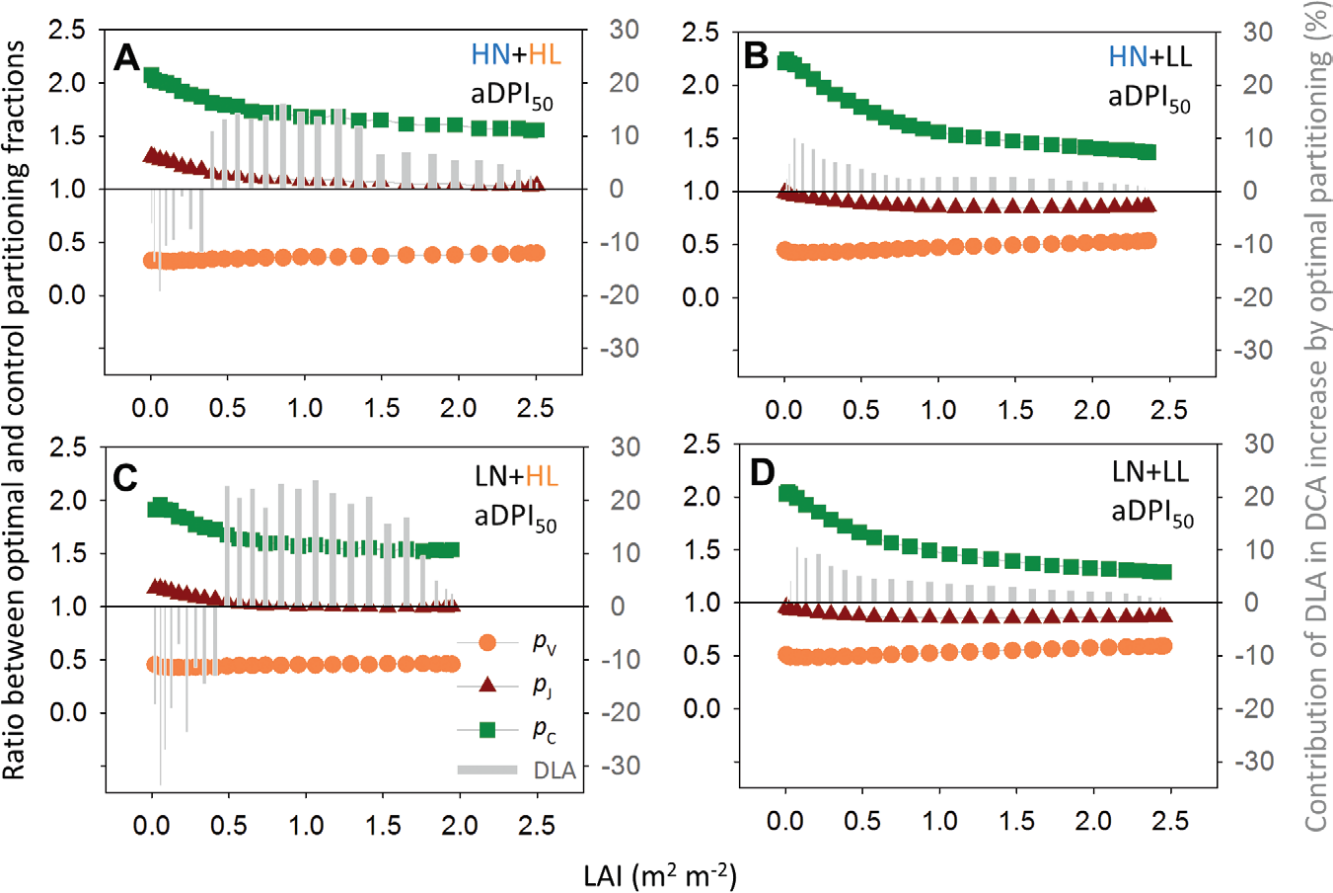
Ratio between optimal and control partitioning fractions (optimal *p*_*X*_/control *p*_*X*_), and contributions of daily leaf carbon assimilation (DLA) to the daily canopy carbon assimilation (DCA) increase by optimal partitioning (grey bars, right *y*-axis) along the canopy depth [leaf area index (LAI) m^2^ m^−2^] under 50% average daily photon integral during acclimation (aDPI_50_) for plants grown under (A) high nitrogen and high light (HN+HL), (B) high nitrogen and low light (HN+LL), (C) low nitrogen and high light (LN+HL), (D) low nitrogen and low light (LN+LL) conditions. Photosynthetic nitrogen partitioning is close to optimum for LN+HL under aDPI_50_, which corresponds to a DPI of 10.7 and 4.3 mol photons m^−2^ d^−1^ for HL and LL, respectively. The symbols and colors used here are the same as those in Fig. 7. See Table 4 for the increase in DCA by the optimal partitioning.

By optimizing *N*_ph_ partitioning, DCA could be increased by nitrogen reinvestment in the limited functional pools. Under aDPI_200_, *N*_ph_ partitioning was suboptimal under HL (Fig. 6), and this suboptimality was less under HN than under LN (Table 4). By reinvesting about half of *N*_C_ into *N*_V_ and *N*_J_ (Fig. 7A, C), DCA increased by 6% under HN and by 13% under LN (Table 4), as a result of increased carbon assimilation in the middle-lower canopy (Fig. 7A, C). Under aDPI_50_, HN did not reduce the suboptimality in *N*_ph_ partitioning (Table 4) due to an underinvestment in the light harvesting function. Reinvesting *N*_V_ into *N*_C_ in the middle or upper canopy (HL, Fig. 8A; LL, 8B, 8D) increased DCA by 7–25% (Table 4).

## Discussion

This model is the first approach applying a dynamic protein turnover mechanism at the leaf level to assess the optimality and limitation in nitrogen use at the canopy level. Here, maximized canopy carbon assimilation is considered as a general indicator of maximizing fitness. The adaptation of the protein turnover mechanism gives reasonable predictions of optimal *N*_ph_ and accurate predictions of leaf photosynthetic traits.

### Mechanistic explanation of leaf nitrogen economics under a wide range of light and nitrogen availabilities

It is well documented that light has the major control of leaf economics. For example, specific leaf area, an integrative indicator of leaf structure that co-varies with leaf nitrogen content (Anten *et al.*, 1998), shows more plastic responses to light than to nutrient availability (Poorter *et al.*, 2009; Poorter *et al.*, 2010). Mechanistic models can be used to interpret measured biological data (Chen *et al.*, 2015, 2018), as in our model here, providing a quantitative explanation of the different plastic responses in leaf nitrogen economics (e.g. photosynthetic nitrogen per unit leaf area, *N*_ph_, and photosynthetic capacities) to light and to nitrogen by their effects on the maximum protein synthesis rate (*S*_max_, Fig. 1). A 5-fold increase in light (4–20 mol photons m^−2^ d^−1^) doubled *S*_max_ of the carboxylation pool (*N*_V_) and electron transport pool (*N*_J_; Fig. 1A, B), which is similar to the published values (Niinemets *et al.*, 2015). In contrast, increasing nitrogen supply from 2 to 10 mM increased *S*_max_ of *N*_V_ and *N*_J_ only by 20% and 16%, respectively. The effects of light on photosynthetic nitrogen can be quantitative (on *N*_ph_) or qualitative (on nitrogen partitioning, *p*_*X*_; Niinemets *et al.*, 2006; Buckley *et al.*, 2013), while nitrogen only affected *N*_ph_ by restricting *S*_max_ (Figs 1, 3). Similar effects of light and nitrogen availabilities on the partitioning between electron transport and light harvesting functions were observed in spinach (Terashima and Evans, 1988). Our model of protein turnover explains the photosynthetic acclimation to light and nitrogen supply and provides a mechanistic insight into leaf nitrogen economics.

In a growing canopy, leaf age is associated with decreasing light availability (Niinemets *et al.*, 2006, Chen *et al.*, 2014). Therefore, leaf photosynthetic acclimation to light occurs together with leaf ageing, which is characterized by the protein degradation constant *D*_r_ and the constant *t*_d_ describing the decrease of protein synthesis rate in our model. The *D*_r_ values of *N*_V_ and *N*_J_ fall within the range of *in vivo* quantifications reported by Peterson *et al.* (1973) and Li *et al.* (2017). The low value of *t*_d_ (Table 1) explains the modest influence of ageing on leaf photosynthetic capacity observed under constant light conditions (Pettersen *et al.*, 2010*a*).

Besides light and nitrogen availability, temperature has effects on photosynthetic nitrogen content and partitioning (Yamori *et al.*, 2005; Kattge and Knorr, 2007; Yamori *et al.*, 2009). Temperature dependency of developmental processes and biochemical reactions is often described by exponential or Arhenius-type functions (Parent *et al.*, 2010; Parent and Tardieu, 2012; Kahlen and Chen, 2015). In our model, temperature effects are considered partly by the temperature sum, which assumes a linear relationship between protein synthesis and leaf temperature. Since the exact temperature dependency of protein synthesis and degradation is unknown and our data are obtained from controlled environments with minimized temperature fluctuations, we apply the linear parsimonious approach to avoid speculation and overparameterization (Parent *et al.*, 2016).

### Above-optimum Rubisco investment can be a mechanism to adapt canopy photosynthesis to short-term light fluctuations

Under sufficient nitrogen availability, Rubisco can function as a storage protein, which means that the amount of Rubisco can exceed the requirements to support photosynthesis (Carmo-Silva *et al.*, 2015). The Rubisco pool has the highest value of *k*_N_ (Table 1), indicating that Rubisco synthesis reacts with higher sensitivity to increasing nitrogen availability than the other two pools. This explains the increase in the ratio between *V*_cmax_ and *J*_max_ with nitrogen availability (Hikosaka, 2004; Yamori *et al.*, 2011*a*), especially under LL (Fig. 3C, E). Under HN, Rubisco storage is advantageous since light-induced Rubisco activation, having a time constant of 3–5 min (Portis *et al.*, 1986; Kaiser *et al.*, 2018), is much faster than Rubisco synthesis. Therefore, Rubisco storage can be a mechanism for quick adaptation to a sudden increase in light. This explains why the plants grown under HN have wider ranges of DPI, at which nitrogen partitioning is optimal, than those under LN (Fig. 6; Table 4). Furthermore, excluding Rubisco activation [*V*_c_=*V*_cmax_ in Eq. (9b)] in the DCA simulation resulted in a 4-fold above-optimum investment in *N*_V_ even under aDPI (data not shown). Since Rubisco is not an especially inefficient catalyst in comparison with other chemically related enzymes (Bathellier *et al.*, 2018), above-optimum Rubisco investment in the canopy can be rather a mechanism for adapting to short-term light fluctuation than a mechanism to overcome its enzymatic inefficiency.

### Implications for crop model improvement and greenhouse management

Using plant models to understand crop performance requires knowledge of physiological mechanisms (Boote *et al.*, 2013; Poorter *et al.*, 2013). By integrating the known biological mechanism of protein turnover at the leaf level into a multi-layer model of canopy photosynthesis, we demonstrate the explanatory power of a mechanistic model for the measured biological data. Our simulations suggest that canopy photosynthesis can be increased by manipulating the functional pools related to photosynthesis. For example, investment in Rubisco and electron transport (Ishimaru *et al.*, 2001; Yamori *et al.*, 2011*b*) should be increased under increasing light (Fig. 7), and a larger antenna size for light harvesting (Masuda *et al.*, 2003) is required under decreasing light availability (Fig. 8). It is clear that the pattern of optimal nitrogen partitioning depends strongly on light regime, and biosynthetic regulation is unlikely to keep up with daily light fluctuation (up to 4-fold difference; Supplementary Fig. S3).

In greenhouse cultivation, it is possible to achieve a more stable light environment using supplemental lighting. This can be a plausible solution to improve the vertical light distribution (Lu and Mitchell, 2016) and to minimize the suboptimality in nitrogen use induced by light fluctuation. Since carbon assimilation is the rate-limiting step for yield production of cucumber plants due to the indeterminate production of vegetative and generative organs (Wiechers *et al.*, 2011), canopy carbon gain can be considered as an approximation for yield. Our simulation suggests that the suboptimal nitrogen partitioning induced by a 50% decrease in DPI can be compensated by reducing the light limitation of the shaded leaves using inter-row lighting during the high-light season (*ca*. 7% increase in DCA) and using top-lighting, possibly in combination with inter-lighting, during the low-light season (*ca*. 25% increase in DCA; Table 4; Fig. 8), similar to the reported increase in cucumber fruit yield (22%–31%) by inter-lighting in the winter season (Kumar *et al.*, 2016). In the summer season, suboptimal nitrogen partitioning induced by sudden doubling in DPI can be overcome by pre-treatment of increasing nitrogen supply and inter-lighting (*ca*. 6% increase in DCA; Table 4), which maintains the biochemical capacity and reduces the biochemical limitation of the shaded leaves (Pettersen *et al.*, 2010*b*; Trouwborst *et al.*, 2010; Chen *et al.*, 2014). These results provide a physiological explanation at canopy level for the observations of supplemental lighting experiments (Hovi *et al.*, 2004; Hovi-Pekkanen and Tahvonen, 2008; Pettersen *et al.*, 2010*b*; Trouwborst *et al.*, 2010). Furthermore, the relationship between protein synthesis rate and intercepted light intensity is non-linear in our model [Eq. (7)], which may offer an explanation why the photoacclimatory responses of a leaf grown under natural within-day light fluctuation differ from that under constant light, as shown in a recent experimental study (Vialet-Chabrand *et al.*, 2017).

Light fluctuations occur particularly in the lower canopy layer, where sunflecks cause strong and frequent variations in light, thereby increasing variations of *N*_ph_ and *p*_*X*_ in the older leaves (Fig. 3). Interestingly, leaves under HL seemed to prioritize their nitrogen investment in *N*_J_ over *N*_C_ under LN with increasing leaf age (Fig. 3E), which might be explained by the reduced LAI development under LN+HL and, hence, the higher light interception of the older leaves (Figs S4C, S5). Since within-leaf and within-day light heterogeneity (e.g. sunflecks) were not described in the model, these variations observed in the greenhouse experiment could not be reproduced in the simulations (Supplementary Fig. S6). This can be improved by coupling the model with a 3D structural plant model and the use of shorter time steps in the simulations to capture more realistic response of photoacclimation.

## Conclusions

We propose a mechanistic model to quantify the effects of leaf age, nitrogen and light availabilities on photosynthetic acclimation. The model predicts the observed photosynthetic acclimation under different combinations of nitrogen supply and light availability in the greenhouse. Model simulation indicates that photosynthetic nitrogen distribution is close to optimum and photosynthetic nitrogen partitioning can be optimal under constant light conditions. However, large fluctuation in light between days under natural conditions inevitably leads to suboptimal nitrogen partitioning. Our study provides insights into photosynthetic acclimation and the model can be used for crop model improvement and provides guidelines for greenhouse management.

## Supplementary data

Supplementary data are available at *JXB* online.

Fig. S1. Schematic diagram of photosynthetic nitrogen turnover.

Fig. S2. Relationship between relative chlorophyll content and leaf chlorophyll concentration.

Fig. S3. Environmental input for the model evaluation and simulation.

Fig. S4. Relationships between leaf angle, LAI, and age.

Fig. S5. Leaf area distribution used as input in the daily canopy assimilation simulation.

Fig. S6. Comparisons of simulated photosynthetic nitrogen traits between nitrogen supply levels and between light conditions.

Fig. S7. Leaf photosynthetic nitrogen distributions with the vertical light distribution.

Table S1. Canopy characteristics used in the daily canopy assimilation simulation.

Supplementary Figures S1-S7 and Table S1

## Abbreviations

aDPI: average daily photosynthetic photon integral during acclimation
Chl: chlorophyll per unit leaf area
DCA: daily canopy carbon assimilation
DPI: daily photosynthetic photon integral
HL: high light
HN: high nitrogen
*J*_max_: maximum electron transport rate
LL: low light
LAI: leaf area index
LN: low nitrogen
*N*_C_: light harvesting pool of photosynthetic nitrogen
*N*_J_: electron transport pool of photosynthetic nitrogen
*N*_ph_: photosynthetic nitrogen
*N*_V_: carboxylation pool of photosynthetic nitrogen
PNUE: photosynthetic nitrogen use efficiency
PPFD: photosynthetic photon flux density
*V*_cmax_: maximum carboxylation rate
