# Supplementary material for: Environmental triggers for photosynthetic protein turnover determine the optimal nitrogen distribution and partitioning in the canopy
Source: J Exp Bot. 2018 Aug 17;70(9):2419–34. doi: 10.1093/jxb/ery308 (PMC6519421; doi:10.1093/jxb/ery308)
Supplement: Supplementary Figures S1-S7 and Table S1 [file ery308_suppl_supplementary_figures-s1-s7_table-s1.pdf]

Supplementary data

RESEARCH PAPER

Environmental triggers for photosynthetic protein turnover determine the optimal nitrogen distribution and partitioning in the canopy

Yi-Chen Pao, Tsu-Wei Chen, Dany Pascal Moualeu-Ngangue, Hartmut Stützel

Institute of Horticultural Production Systems, Leibniz Universität Hannover, Hannover, Germany

Yi-Chen Pao

Email: pao@gem.uni-hannover.de

Telephone: +49 511 762-19269

Fax: +49 511 762-3606

This work was carried out at the Institute of Horticultural Production Systems, Leibniz Universität Hannover.

Date of submission: 27 March 2018

Table S1. Canopy characteristics used in the daily canopy carbon assimilation (DCA) simulation for the plants grown under combinations of high nitrogen (HN), high light (HL), low nitrogen (LN) and low light (LL). Measured leaf area (LA, m^2), estimated thermal age (*t*, °Cd) and simulated elevation angle (ε, °) are listed for each leaf rank. Cumulative leaf area index of the canopy (cLAI, m^2 m^{-2}) and total leaf photosynthetic nitrogen content in the canopy (N_{canopy}, mmol N) were used as input in the DCA simulations. *t* was estimated using a constant phyllochron (°Cd) under each growth condition since significant differences were found between conditions ($P < 0.001$). ε was simulated using the empirical relationship with *t* (Fig. S4A and S4B).

Growth condition	HN+HL			HN+LL			LN+HL			LN+LL		
Leaf rank	LA m²	*t* °Cd	ε °	LA m²	*t* °Cd	ε °	LA m²	*T* °Cd	ε °	LA m²	*t* °Cd	ε °
1	0.0298	566	-32	0.0157	548	-35	0.0288	566	-32	0.0176	548	-35
2	0.0610	540	-36	0.0365	522	-38	0.0482	540	-36	0.0512	522	-38
3	0.0863	514	-39	0.0525	496	-42	0.0651	514	-39	0.0519	496	-42
4	0.1046	496	-42	0.0597	480	-44	0.0807	494	-42	0.0634	478	-44
5	0.1181	479	-44	0.0673	464	-46	0.0914	473	-45	0.0850	460	-46
6	0.1093	461	-46	0.0752	448	-48	0.0905	453	-47	0.0914	442	-49
7	0.1278	443	-49	0.0777	432	-50	0.0894	433	-50	0.0935	424	-51
8	0.1247	425	-51	0.0870	416	-52	0.0878	412	-53	0.0925	406	-54
9	0.1077	408	-53	0.0983	400	-54	0.0844	392	-55	0.0940	388	-56
10	0.0998	390	-56	0.0917	384	-56	0.0840	372	-58	0.0986	370	-58
11	0.0992	372	-58	0.0974	368	-58	0.0840	352	-60	0.1036	352	-60
12	0.0831	355	-60	0.0935	352	-60	0.0760	331	-62	0.1042	334	-62
13	0.0858	337	-61	0.0874	335	-62	0.0630	311	-64	0.0996	316	-63
14	0.0839	319	-63	0.0838	319	-63	0.0645	291	-65	0.0970	298	-64
15	0.0715	302	-64	0.0766	303	-64	0.0593	270	-66	0.0962	280	-65
16	0.0672	284	-65	0.0663	287	-65	0.0578	250	-66	0.0896	262	-66
17	0.0635	266	-66	0.0563	271	-66	0.0524	230	-65	0.0843	244	-66
18	0.0596	248	-66	0.0571	255	-66	0.0439	209	-64	0.0736	226	-65
19	0.0520	231	-65	0.0592	239	-66	0.0426	189	-61	0.0690	208	-64
20	0.0532	213	-64	0.0605	223	-65	0.0401	169	-56	0.0682	190	-61
21	0.0424	195	-62	0.0639	207	-64	0.0361	149	-50	0.0607	172	-57
22	0.0407	178	-59	0.0633	191	-61	0.0302	128	-41	0.0616	154	-52
23	0.0363	160	-54	0.0561	174	-58	0.0235	108	-28	0.0441	136	-45
24	0.0304	142	-47	0.0504	158	-53	0.0252	88	-12	0.0311	118	-35
25	0.0281	125	-39	0.0521	142	-47	0.0158	67	10	0.0127	100	-23
26	0.0131	107	-27	0.0402	126	-40	-	-	-	0.0073	82	-7
27	0.0050	89	-13	0.0244	110	-30	-	-	-	0.0044	64	13
28	-	-	-	0.0133	94	-17	-	-	-	-	-	-
29	-	-	-	0.0089	78	-2	-	-	-	-	-	-
30	-	-	-	0.0036	62	17	-	-	-	-	-	-
N_{canopy} (mmol N)	95.84			68.29			69.24			62.90		
cLAI (m^2 m^{-2})	2.506			2.362			1.948			2.455		
Phyllochron (°Cd)	17.7			16.1			20.3			18.0		

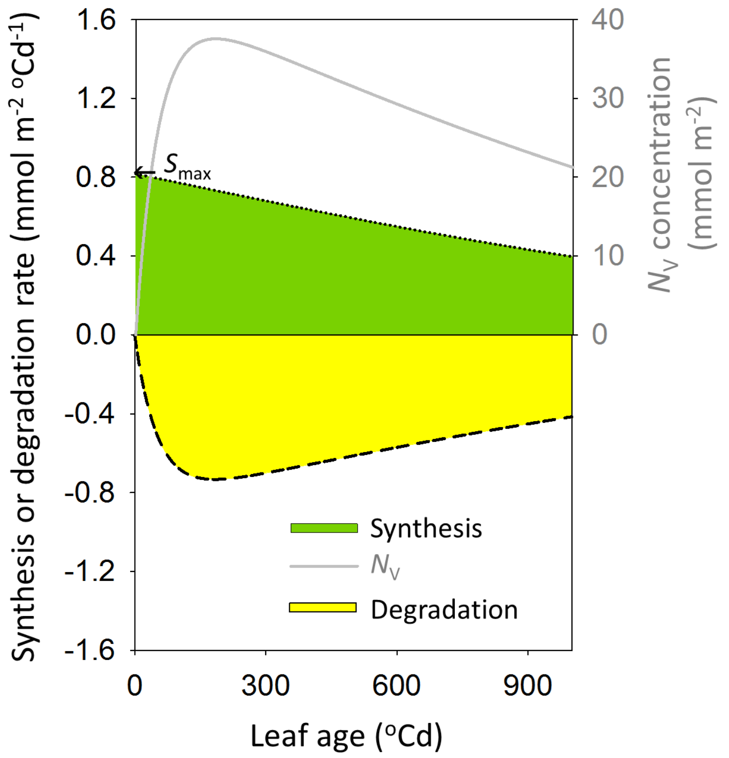

Fig. S1. Schematic diagram of the dynamics of photosynthetic nitrogen with leaf age. The carboxylation nitrogen pool [N_V, mmol N m^{-2}, grey solid line, Eq. (4)] is shown here as an example, which was determined simultaneously by the synthesis rate [mmol N m^{-2} °Cd^{-1}, dotted line filled with green shade, Eqs. (5), (7) and (8)] and the degradation rate [mmol N m^{-2} °Cd^{-1}, dashed line filled with yellow shade, Eq. (6)]. The coefficients were taken from Table 1. Constant light level of 21.4 mol d^{-1} m^{-2} and nitrogen supply level of 10 mM were used in this simulation. Photosynthetic nitrogen pools exhibited the similar evolutionary pattern with leaf age under constant light in the growth chamber experiment, first increasing to a maximum (at 150-250°Cd) and then decreasing as leaf aged.

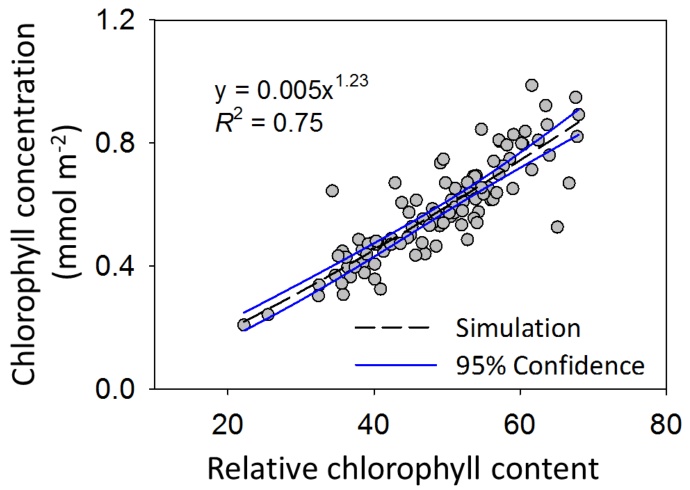

Fig. S2. Relationship between relative chlorophyll content (SPAD) and leaf chlorophyll concentration (*Chl*, mmol Chl m^{-2}) for non-destructive estimation of *Chl* in the greenhouse experiment. The dashed line indicates the simulated relationship between the relative chlorophyll content and *Chl* with 95% confidence interval (blue solid line).

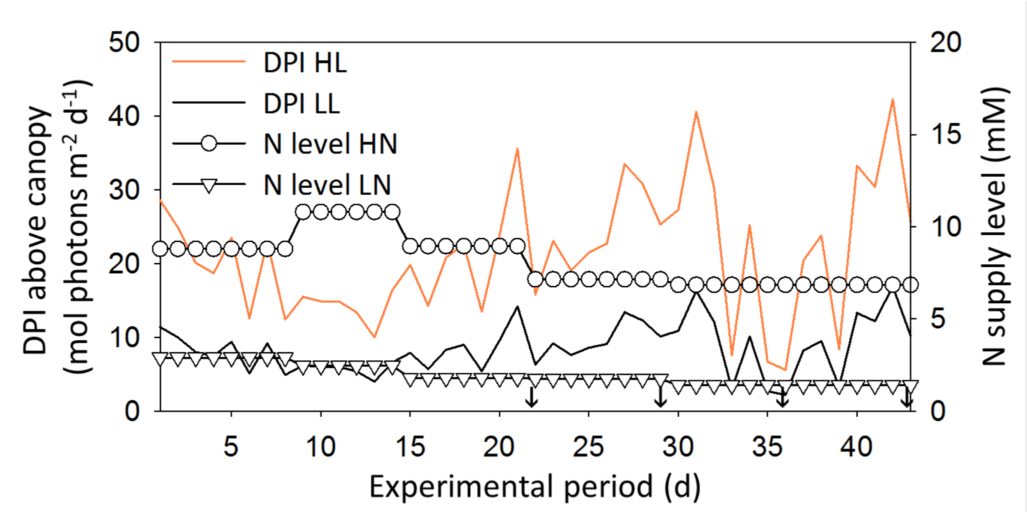

Fig. S3. Environmental data input for model evaluation and simulation. Daily photon integral (DPI, mol photons m^{-2} d^{-1}) recorded during the greenhouse experiment by the weather station located outside of the greenhouse. Light transmittance through greenhouse of 49.8% was applied to estimate the DPI above the canopies in the greenhouse for high growth light (HL, orange solid line). The DPI was further reduced to 40% of HL by shading under the low growth light (LL, black solid line). The nitrogen supply level in nutrient solution (mM, right y-axis) was analyzed weekly for high nitrogen (HN, open circles) and low nitrogen (LN, open triangles). Four measurement dates are indicated by the arrows.

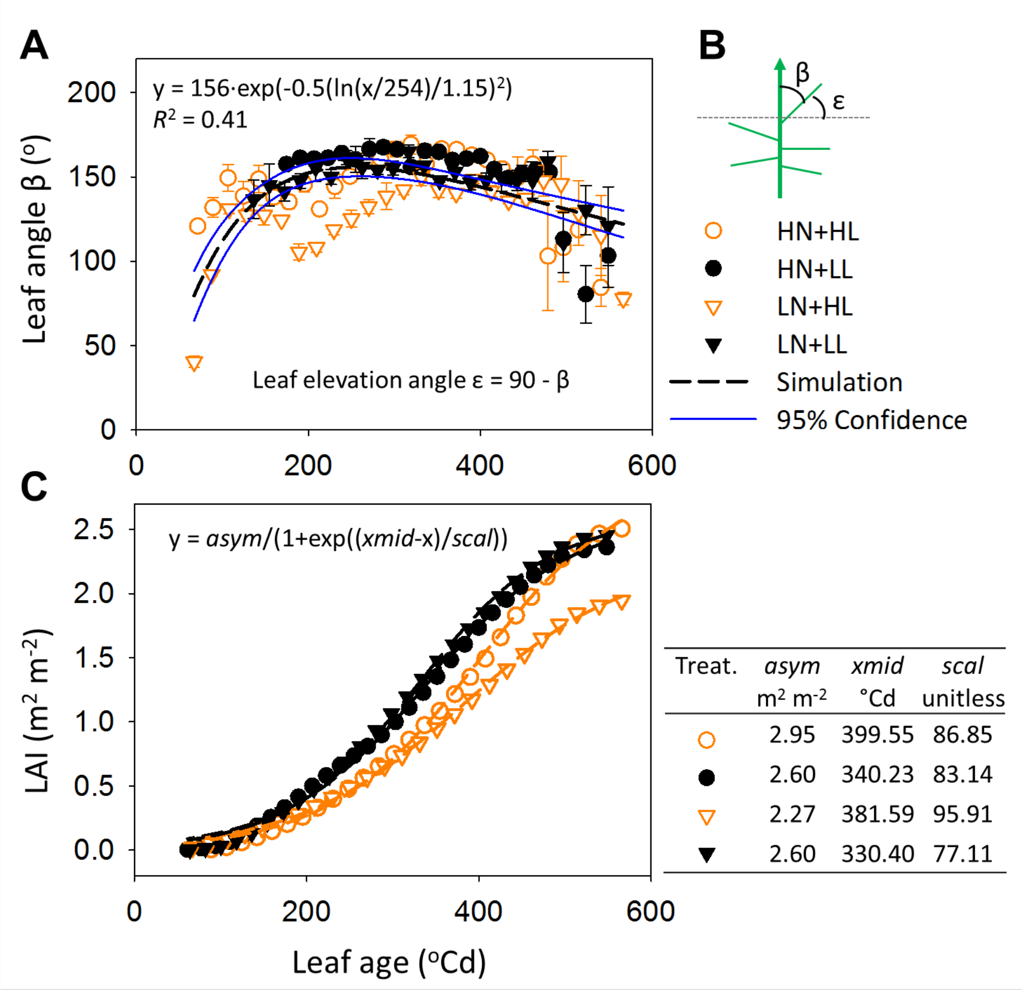

Fig. S4. Relationships between leaf age (t, °Cd) and the leaf characteristics applied in the dynamic protein turnover model for simulating local light intensity at the leaf. (A) Relationship between t and leaf angle β (°); the dashed line indicates the simulated values with 95% confidence interval (blue solid line). (B) Leaf angle β was defined as the angle between the line from the base to the tip of a leaf and the vertical plane. Leaf elevation angle (ε, Table S1) was converted as ε = (90 - β). (C) The logistic relationship between t and leaf area index (LAI, m^2 m^{-2}); the dashed lines indicate the simulated values and the coefficients used for each treatment are listed. Measured values of different treatments are shown as: high nitrogen and high light (HN+HL, orange open circles), high nitrogen and low light (HN+LL, black closed circles), low nitrogen and high light (LN+HL, orange open triangles), low nitrogen and low light (LN+LL, black closed triangles).

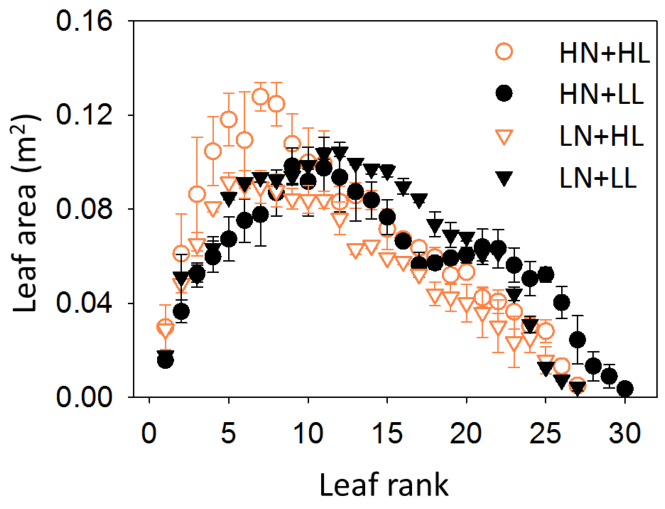

Fig. S5. Leaf area (m^2) distribution used as input in the daily canopy carbon assimilation simulation for plants grown under different conditions. The symbols and colors used here are the same as those in Fig. S4.

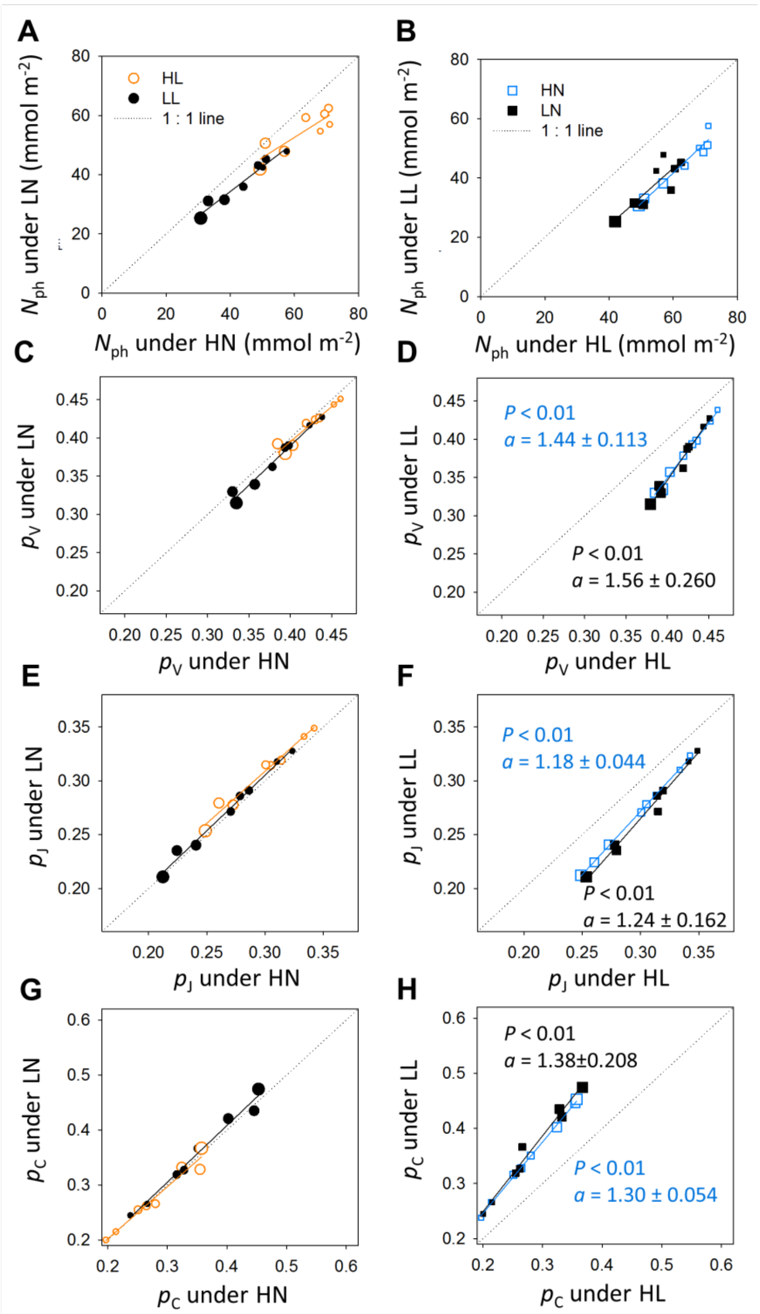

Fig. S6. Comparisons of simulated leaf photosynthetic nitrogen (N_{ph}, mmol N m^{-2}, A and B), partitioning fractions of the carboxylation pool (p_V, C and D), the electron transport pool (p_J, E and F) and the light harvesting pool (p_C, G and H) between high and low nitrogen supply (HN and LN, respectively, A, C, E and G) and between high and low light conditions (HL and LL, respectively, B, D, F and H). Each point represents the simulations obtained from a comparable canopy layer as those in Fig. 2. The orange open circles indicate leaves grown under HL, the black closed circles indicate LL, the blue open squares indicate HN and the black closed squares indicate LN. The size of the circles increases with leaf age (ranging from 77˚Cd to 414˚Cd). The solid lines show the linear regression y = *ax* + *b*. The values of *a* are specified with 95% confidence intervals when they are significantly different from one. The dotted grey lines denote one-to-one lines.

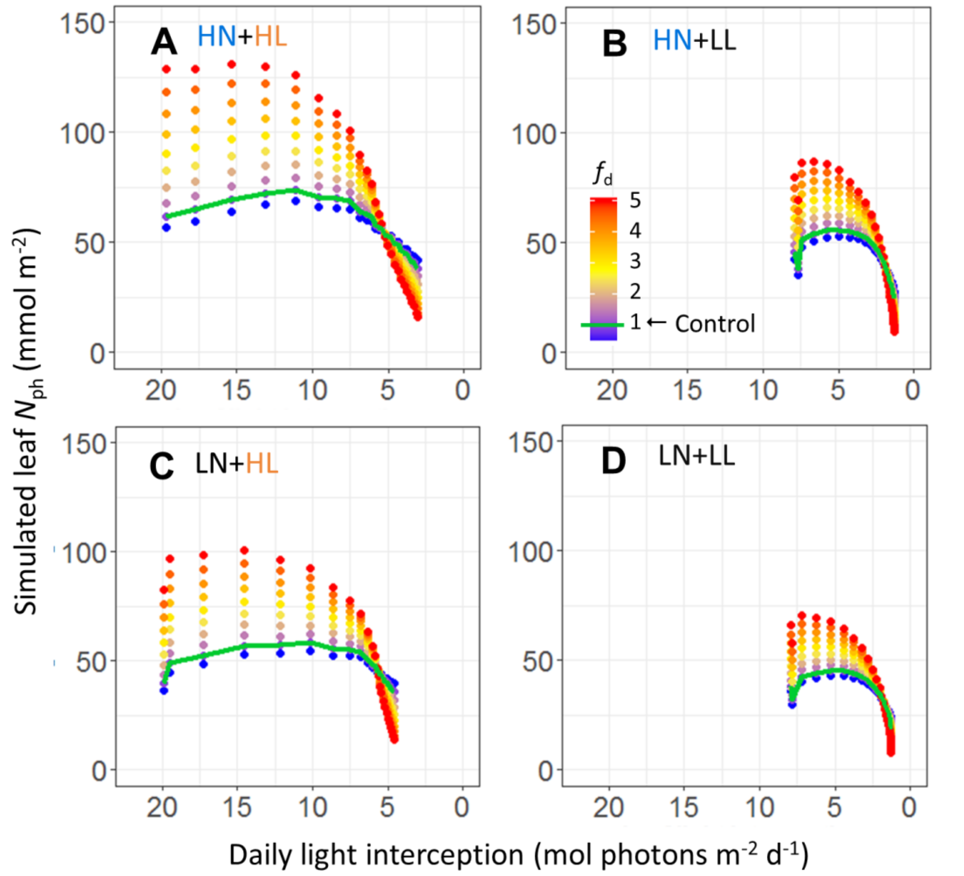

Fig. S7. Leaf photosynthetic nitrogen (N_{ph}, mmol N m^{-2}) distributions with the vertical light distribution, characterized by daily light interception (mol photons m^{-2} d^{-1}). The variations in nitrogen distribution were created using a distribution factor f_d ranging from 0.5 to 5.0 at intervals of 0.5 in Eq. (19) under different growth conditions. (A) High nitrogen and high light (HN+HL); (B) high nitrogen and low light (HN+LL); (C) low nitrogen and high light (LN+HL); (D) low nitrogen and low light (LN+LL). Simulated control N_{ph} distributions (f_d = 1) are indicated by the green lines.
